# Supplementary material for: HOXA11-As Promotes Lymph Node Metastasis Through Regulation of IFNL and HMGB Family Genes in Pancreatic Cancer
Source: Int J Mol Sci. 2024 Nov 30;25(23):12920. doi: 10.3390/ijms252312920 (PMC11641524; doi:10.3390/ijms252312920)
Supplement: Supplementary file 1 [file ijms-25-12920-s001.zip › Suppl Tables_20241117.pdf]

Supplementary Table S1. Genes upregulated in PDAC with lymph node metastasis

Gene Name	Fold Change (log2)
ACTG1P11	3.41977138
CYCSP45	3.402007406
CSAG3	3.258402944
RPL7P56	3.156191573
RPL21P133	3.154917069
RP11-308B5.2	3.135666275
DCAF12L1	3.127073377
GS1-421I3.2	3.050410115
AKAP14	3.046796638
RP13-347D8.1	3.045635387
Y_RNA.771	2.97982846
KCTD9P2	2.979396236
LL0XNC01-116E7.5	2.955248736
GLRA4	2.933621438
RP5-961K14.1	2.920319338
INGX	2.91827672
RP3-323B6.1	2.911566025
XAGE3	2.898816147
RN7SL262P	2.870099663
AF196972.3	2.820505684
MIR221	2.819609848
HYPM	2.812658459
AL121578.2	2.809213609
SNRPEP9	2.786523026
RP11-1M18.1	2.738011285
RP11-706O15.7	2.717717985
RP11-558O12.1	2.715373663
RNA5SP498	2.706927671
BX649553.2	2.693576122
CTA-384D8.31	2.690952931
RP1-32I10.10	2.685464799
RP4-742C19.12	2.664103135
MIR6819	2.63273741
MIR3909	2.63066235
RP3-438O4.4	2.62783333
CTA-243E7.3	2.615611544

PRAME	2.615390662
IGLV7-35	2.608024366
IGLV5-52	2.602044677
IGLVI-70	2.589560795
AC006946.12	2.574142173
AP001065.7	2.567088958
MIR6814	2.562204242
LINC00114	2.559321299
LINC01423	2.559154318
AP001434.2	2.558105363
AJ006998.2	2.537776423
CBS	2.530485539
GATA5	2.523610298
SNORD12C	2.521131963
RBPJL	2.52047972
FAM83C	2.516741976
LINC00028	2.514520185
RNU6ATAC17P	2.489339499
GGTLC1	2.479021938
RP4-775C13.1	2.477394179
SNORA31.24	2.468858984
VN2R19P	2.460004679
AC010525.1	2.455705318
AC008746.10	2.445089021
AC008984.6	2.443945765
AC010518.2	2.440670614
SIGLEC18P	2.437821427
KLK7	2.434541547
KLK1	2.434108238
AC010524.4	2.427750772
KCNA7	2.42729265
CGB5	2.424412188
CTB-60B18.6	2.41611857
CGB	2.409408886
ERICH4	2.403058079
RN7SL34P	2.393437565
AC092071.1	2.380489134
IFNL3P1	2.372772248
SYCN	2.364928938

LGALS7	2.361602392
OVOL3	2.356856668
AC079466.1	2.354422169
AC005307.3	2.342296228
AC005307.1	2.336573029
RP11-255H23.5	2.334113098
LINC01224	2.324952564
ZNF209P	2.318147421
AC025811.3	2.317270807
BNIP3P28	2.315308472
VN1R84P	2.308471532
CTD-2561J22.1	2.305111374
CTD-2332E11.2	2.304331583
CTC-513N18.6	2.299865415
AC006539.1	2.292173577
AC007204.2	2.289281719
CTC-412M14.6	2.284789996
AC005796.2	2.283581874
RSL24D1P8	2.28234205
MBD3L1	2.281652633
RP11-1137G4.3	2.281102494
AC005624.2	2.2796384
RNA5SP462	2.277762138
FAM60CP	2.273086462
RNU2-69P	2.264437062
RP11-958F21.1	2.263739403
RNA5SP452	2.26145693
RNU5A-6P	2.261052022
Y_RNA.674	2.25886898
RP11-699A5.2	2.252581963
RHOT1P1	2.248070579
SDHDP1	2.247331062
ANKRD62	2.243368777
RP11-703I16.3	2.236453429
Y_RNA.669	2.224443098
UTS2R	2.220103097
RP11-1055B8.2	2.207206529
MIR657	2.202581896
Y_RNA.661	2.201012149

CTD-2006K23.2	2.200362848
AC079325.6	2.197076609
MIR4524B	2.195545862
AC025048.1	2.194901037
RNF126P1	2.190891386
RP11-304F15.7	2.189920582
GIP	2.189341564
MIR152	2.188394047
RNU7-186P	2.18780411
Metazoa_SRP.245	2.18650902
RP11-259G18.3	2.185144384
RP11-798G7.5	2.184909622
KRT36	2.177834833
KRTAP4-1	2.173616189
KRT223P	2.173063003
MIR6867	2.17261499
YWHAEP7	2.170159271
CTC-507E2.2	2.169212779
SPACA3	2.169133861
FOXN1	2.168650316
CTB-96E2.10	2.166893577
SCDP1	2.162527069
SRP68P3	2.160314337
RP11-524F11.3	2.159986179
RP11-104H15.10	2.157306945
RP11-459C13.1	2.155559207
RP11-667K14.14	2.154302045
AC144836.1	2.153916797
AC087392.1	2.14992686
FOXC2-AS1	2.149348233
RPS3P7	2.147027838
RP11-538I12.3	2.145611429
RP11-77K12.3	2.141611081
CTRB1	2.133828013
CTRB2	2.132276232
RNU6-208P	2.12759461
RP11-343C2.10	2.125599718
MT1H	2.124240443
CTD-3032H12.1	2.122619329

RP11-327F22.6	2.121493268
AQP8	2.117904922
MIR1273H	2.117024162
CHP2	2.116788432
CTD-2385L22.1	2.115359602
CTD-2196E14.8	2.109761636
AC106788.1	2.109092523
CTD-2547E10.3	2.105243215
GP2	2.103572679
CLEC19A	2.094126954
CTA-276F8.2	2.092751798
RNU1-22P	2.092454303
PRR35	2.090110911
AC107977.1	2.089299202
RP11-66B24.5	2.084602549
CERS3-AS1	2.083589192
RP11-327J17.1	2.08316938
RP11-327J17.2	2.082456589
CTD-2315E11.1	2.081549401
MIR3174	2.07971266
RP11-343B18.2	2.074531621
RP11-2E17.2	2.06827367
RP11-272D12.2	2.065886578
RP11-2I17.4	2.06576964
RP11-352D13.5	2.065141861
RP11-352D13.6	2.058391282
RP11-798K3.2	2.057746409
RP11-330L19.2	2.052519524
RP11-745A24.1	2.049776631
AC092755.4	2.049325645
AC025918.1	2.047635534
RP11-355N15.1	2.046532742
CTD-2515H24.4	2.040258793
snoU13.24	2.039106224
RP11-5N19.3	2.038885645
RP11-643A5.3	2.037025868
RP11-108K3.2	2.035591772
RP11-507J18.5	2.024799768
RNA5SP395	2.023384439

GATM-AS1	2.021733631
CTD-2651B20.4	2.021633417
Metazoa_SRP.207	2.021002979
RP11-323I15.3	2.020369497
RP11-3D4.4	2.019444814
ULK4P3	2.018449842
MIR4715	2.016998705
SNORD116-4	2.016971068
IGHV2-70	2.016629021
IGHVIII-67-2	2.01581308
IGHV3-37	2.014818752
IGHV3-6	2.014351224
AL122127.25	2.014207401
AC246787.3	2.013682291
AL122127.2	2.012784068
U3.36	2.012653113
RP11-638I2.9	2.012201808
RP11-638I2.2	2.009926285
RP11-725G5.2	2.009501105
RP11-356K23.1	2.009276314
AL136040.1	2.008189281
ZMYND19P1	2.007659245
RN7SL356P	2.005492131
AC007182.6	2.004677024
RP6-114E22.1	2.004001095
SNORD56B	2.00029905
PTTG4P	1.998039603
RP6-65G23.5	1.997808206
RP11-204K16.1	1.996411255
CTD-2566J3.1	1.994744723
HIF1A-AS1	1.994025495
AL162759.1	1.992662251
RNU6-301P	1.989894591
RP11-255G12.2	1.987231544
CTD-2298J14.2	1.986594535
RP11-356O9.2	1.986567987
TRAJ11	1.986158548
TRAJ13	1.986112541
TRAJ14	1.98158168

TRAJ31	1.981498839
TRAJ38	1.978459587
TRAV38-1	1.978215338
TRAV35	1.975564077
TRAV26-2	1.973776993
TRAV30	1.972166187
TRAV27	1.969834641
TRAV18	1.965078246
TRAV8-5	1.960769338
TRAV8-1	1.960668967
RPL4P1	1.95988603
TRAV3	1.958722799
EIF4EBP1P1	1.95870273
RP11-146E13.4	1.952816923
RP11-391H12.8	1.952075381
RNA5SP39	1.951139922
FARP1-AS1	1.950502719
LINC00434	1.949553075
RNA5SP29	1.94733837
PCNPP5	1.94671519
RNU6-57P	1.945667099
STOML3	1.944694887
RP11-16D22.2	1.944003965
LINC00544	1.942673851
uc_338.25	1.942009858
TATDN2P3	1.941704086
SGCG	1.939766079
FNTAP2	1.939701223
RP13-672B3.5	1.937194976
RP11-989F5.3	1.936579351
NDUFA5P6	1.936016959
LHX5	1.935508558
SLC25A3P2	1.933529299
RP11-818F20.5	1.932721677
PMCH	1.929305462
RNU6-247P	1.929195315
RP11-753N8.1	1.929013324
SLC6A15	1.928558741
RP11-498M15.1	1.927975279

CTD-2021H9.3	1.926960782
CTD-2021H9.2	1.924689063
FAHD2P1	1.923406466
RP11-159A18.1	1.922860318
RP11-611O2.2	1.920563571
NTAN1P3	1.920475824
RP11-335I12.2	1.917125538
RP11-715H19.2	1.913829035
RP11-813P10.2	1.905489654
RP11-58A17.4	1.905191088
MIR148B	1.90287608
HOXC11	1.902618499
HOTAIR	1.900921427
RP1-90J4.1	1.900214393
RP11-493L12.6	1.898101772
RP11-493L12.3	1.89574387
MIR4698	1.894534116
RP11-946L16.2	1.894049808
RP11-662I13.2	1.892796804
CTC-465D4.1	1.891588442
RP11-841C19.1	1.889929725
RP11-459D22.1	1.889242987
LINC01559	1.888288549
RP11-113C12.4	1.887722065
NANOGP1	1.887314491
RP5-940J5.8	1.886287796
RP11-320N7.2	1.882170471
LINC00940	1.880494671
RP11-288K12.1	1.880147766
AP002856.5	1.88002492
AP002954.6	1.878610725
RNU6-1157P	1.877818119
APOA4	1.876239663
RP11-89C3.3	1.875242358
SLN	1.875041053
AP001282.1	1.873335016
RP11-680E19.2	1.869126059
RP11-680E19.1	1.868597873
CARD17	1.86477174

RP11-617B3.2	1.862311328
RP11-563P16.1	1.859751424
RP11-690D19.4	1.859223617
RNU7-159P	1.856623093
BOLA3P1	1.85474047
RP11-817J15.3	1.852186887
RP11-727A23.8	1.85181558
CTD-2337I7.1	1.851642422
RNU6-216P	1.847086005
AP002381.2	1.846516386
RP11-807H22.6	1.843664076
RP11-807H22.7	1.843593286
AP001271.3	1.842667835
CTA-797E19.1	1.839585838
RP11-805J14.3	1.838741865
AP000807.2	1.837060859
GPR152	1.83654464
C11orf86	1.834539959
RP11-867G23.2	1.833827361
RP11-1167A19.2	1.832238328
SPDYC	1.829613734
GPHA2	1.829151358
RP11-697H9.2	1.828729232
IMMP1LP1	1.827361713
SCGB1D2	1.827002759
RP11-1036E20.7	1.826766193
CTD-2560E9.3	1.824824338
RP11-313M3.1	1.824264286
RP11-22P4.2	1.821346561
MUC15	1.821118096
RP11-613F22.6	1.819685772
AC116533.4	1.81857554
CTD-2381F24.1	1.818164358
RP11-351I24.3	1.815518155
OR52E8	1.814845241
OR52V1P	1.813355034
Y_RNA.455	1.810844562
RP11-326C3.13	1.81030214
IFITM5	1.810120348

RP11-234G16.5	1.80759513
RP11-47G11.2	1.805963457
ZNRD1-AS1_2.1	1.805951273
PNLIPRP2	1.80523891
PNLIPRP1	1.803914291
PNLIP	1.802446855
RNU6-1165P	1.801184532
CPN1	1.800763109
RNA5SP323	1.799998543
LIPN	1.79928946
NPAP1P3	1.798052581
OPN4	1.796863635
C10orf99	1.794831935
RP11-40F6.1	1.79474806
RP11-472G21.2	1.793316241
MIR7152	1.792905772
RP11-227H15.7	1.791023086
Metazoa_SRP.140	1.790821524
LINC01468	1.786879384
RP11-346D6.4	1.786283646
CHAT	1.78557622
HNRNPA1P33	1.783499824
RP11-733D4.2	1.783176955
AL022345.10	1.782915936
RP11-810B23.1	1.781897262
PTF1A	1.781500764
SNRPGP5	1.780448799
RP3-323N1.2	1.780157093
RP11-554I8.1	1.777309462
Y_RNA.418	1.777006937
CALML5	1.775679293
RP11-885N19.6	1.775503557
RP11-473E2.4	1.774251397
CELP	1.773272143
CEL	1.772559401
OR1J4	1.771824862
RP11-542K23.9	1.767782628
RP11-542K23.7	1.767771569
RP11-168K11.2	1.767061755

MIR4668	1.765763405
RP11-569G13.3	1.764344761
NUTM2F	1.762924903
RPS6P12	1.762693373
RP11-157L3.6	1.762178964
RP11-392E22.11	1.761841987
CNTFR-AS1	1.761813671
RP11-537H15.4	1.760642415
AC139103.1	1.759810435
CCDC26	1.759530885
CASC19	1.759195917
KNOP1P5	1.758204494
RN7SL329P	1.755364003
RP11-1101K5.1	1.754859085
RP11-35G22.1	1.754022279
RP11-642D21.1	1.753789488
KB-1991G8.1	1.750657945
RPS23P1	1.750128786
RP11-257P3.3	1.748002904
RP11-956J14.1	1.744122988
RP11-17A4.2	1.74308581
RP11-1081M5.3	1.741778455
ASNSP1	1.741131136
RNU1-124P	1.74076465
Y_RNA.366	1.740010215
CHRNB3	1.737829608
RP11-1007J8.1	1.73773734
U6.10	1.73768989
RP11-51J9.4	1.736132565
RP11-486M23.2	1.735086717
CTD-2647L4.1	1.73496308
RP11-213G6.2	1.73442243
RP11-875O11.3	1.734155939
RP11-582J16.3	1.734099103
RP11-1105O14.1	1.732832946
RP11-108A14.1	1.732703228
MIR8055	1.731671283
EN2	1.730508473
FABP5P3	1.730304292

RP4-669B10.3	1.730054205
RN7SL535P	1.726219755
C7orf34	1.726141337
PRSS3P2	1.725825372
PRSS1	1.724684316
TRBV21-1	1.723631436
TRBV12-3	1.721642981
TRBV13	1.718971755
TRBV11-1	1.718344635
TRBV4-2	1.717714322
TRBV7-1	1.716722931
MOXD2P	1.715552966
ERHP1	1.715457382
STRA8	1.712129935
AC093106.5	1.709952225
CPA1	1.709749861
CPA2	1.70937003
AC000111.5	1.709057896
AC000111.4	1.709033887
AC002543.2	1.708983131
AC002066.1	1.708391817
MUC17	1.708329585
AC002076.10	1.706562437
GNGT1	1.706231832
AC005009.1	1.705319252
AC073850.6	1.705164312
GNAT3	1.704036664
AC007000.10	1.703951097
AC004980.11	1.703355995
TRIM50	1.701767495
AC006480.1	1.700826365
AC007349.5	1.699851173
AC007349.4	1.698311949
Y_RNA.319	1.696099794
TBX20	1.695585041
AC006380.3	1.695282466
MIR550A3	1.694030085
RNU6-979P	1.691653923
AC073150.6	1.689485711

AC004009.3	1.688910313
HOTTIP	1.686781545
HOXA13	1.68602952
HOXA11-AS1_5	1.685769286
RP1-170O19.14	1.684023019
HOXA11-AS	1.683202549
HOXA10-AS	1.682126591
HOXA10-HOXA9	1.681914545
CTA-242H14.1	1.681490999
PCMTD1P3	1.680475955
AC079780.3	1.680420509
AC099342.1	1.67998131
RP4-594A5.1	1.67846683
RP11-730B22.1	1.677736352
AC226118.1	1.677469519
RP3-495K2.2	1.676947978
RP3-393E18.3	1.676946455
U8.8	1.675272654
RP11-317B3.2	1.673728772
RP3-352A20.1	1.672491858
RNA5SP217	1.668462394
RP3-425C14.6	1.668171871
RP11-57K17.1	1.668155424
NUDT19P3	1.668110199
RPL35P3	1.667335742
OOEP	1.667204265
MIR30C2	1.667162683
RP11-462G2.1	1.666696362
RP11-524H19.2	1.666154535
TINAG	1.666015577
GSTA2	1.663212193
GSTA7P	1.663051881
RP3-335N17.2	1.659393849
RP3-417L20.4	1.658440037
CRISP2	1.658264513
RP1-229K20.5	1.657725815
BTBD9-AS1	1.65742535
Z85986.1	1.656019417
CLPS	1.655059152

CLPSL1	1.654884037
SNORA40.8	1.654525034
LINC00336	1.654381511
Y_RNA.280	1.653523727
XXbac-BPG248L24.10	1.653453886
DPCR1	1.653427527
TMPOP1	1.653169505
XXbac-BPG170G13.32	1.653074393
LINC01556	1.652935663
OR2B6	1.651564158
HIST1H1B	1.650684058
HIST1H2AI	1.649682022
HIST1H2APS3	1.649522024
LARP1P1	1.648663315
RN7SL334P	1.648487186
AL136303.1	1.648295975
RNU6-190P	1.648066411
RP11-330A16.1	1.647757429
RP11-637O19.2	1.646070832
RP1-182O16.1	1.645728455
BTNL3	1.645658438
CTC-573N18.1	1.643652892
AACSP1	1.642965216
CTD-2532K18.2	1.641925049
LINC01187	1.640678943
FOXI1	1.640249117
HAND1	1.639277768
NMUR2	1.637975605
RPS20P4	1.637892468
CTD-2062A1.2	1.637734326
CTB-161M19.1	1.636736191
RP11-526F3.1	1.636410939
HMGN1P13	1.635688174
GJA1P1	1.635266282
RP11-138J23.1	1.634334285
RNA5SP187	1.632706551
RPL7P24	1.632020969
RPS2P24	1.629350594
CTD-2372A4.1	1.629073742

CTC-537E7.3	1.628019825
CTD-2023N9.1	1.627715147
AC022431.2	1.62637553
CTC-236F12.4	1.625945382
AC008391.1	1.62506285
CTD-2353F22.2	1.624438976
RP11-1C1.4	1.624152963
RP11-1C1.6	1.623317031
RP11-531A21.2	1.622902565
AC091849.1	1.621511179
WWC2-AS1	1.62066895
GTF2F2P1	1.620571086
HSPD1P5	1.620408981
LSM3P4	1.62031623
AC113617.1	1.619239533
AC107399.2	1.619233644
EGF	1.618442978
RP11-710F7.2	1.617517668
RP11-328K4.1	1.616619286
RP11-763F8.1	1.615237769
RNU1-36P	1.614846054
RP11-529H2.2	1.61338131
AMTN	1.612505968
RP11-468N14.13	1.612350879
RP11-468N14.7	1.611556504
RP11-468N14.6	1.609457021
RP11-468N14.5	1.609103096
CTD-2005D20.1	1.608787295
RP11-1267H10.4	1.608308986
CWH43	1.606142959
RP11-109E24.1	1.605492053
RP11-632F7.3	1.604704582
RP11-734I18.1	1.604284815
RP11-206P5.2	1.60379128
FGFBP1	1.603489862
RNA5SP154	1.602343486
RP11-1396O13.1	1.601889459
AC104650.2	1.601620374
LINC01587	1.600982495

RN7SKP275	1.600085593
TMED11P	1.599207459
AC069257.8	1.598524049
MIR6829	1.598465889
RNU6ATAC24P	1.597593638
OPA1-AS1	1.596772975
OSTN	1.59319627
RN7SKP296	1.59207093
GCNT1P3	1.591468438
TPRG1-AS2	1.58918336
RTP2	1.588795713
ADIPOQ	1.58828488
HTR3E	1.587338615
HTR3C	1.586804053
RP11-225N10.3	1.58537092
RP11-646E18.4	1.585140484
RP11-259P15.4	1.584603629
RP11-420J11.1	1.584120038
snoU13.8	1.583585987
ASS1P7	1.581958286
RP11-816B4.1	1.58084242
RP11-408H1.3	1.580781733
SERPINI2	1.580408319
SI	1.580003064
RNU6-1098P	1.579883895
RP11-206M11.7	1.57948436
CPB1	1.578365487
RNU1-100P	1.578052146
RP11-789L4.1	1.578014113
TDGF1P6	1.577723075
RP11-91K8.4	1.577360358
RN7SL752P	1.577024126
RPS15AP16	1.576133934
ALDH1L1-AS2	1.576133082
MIR544B	1.575839772
RPL7P15	1.575308492
OR7E100P	1.574596116
RP11-231E6.1	1.574558832
GUCA1C	1.573495717

RETNLB	1.572527947
RP11-454H13.1	1.57247084
RP11-314M24.1	1.571905779
LSP1P2	1.571878887
RP11-803B1.2	1.571445444
MYLKP1	1.571031565
RP4-555D20.1	1.57070066
AC096921.2	1.570285471
MINOS1P3	1.570240921
Metazoa_SRP.54	1.569771457
RNU6-377P	1.568569795
DUSP5P2	1.567566872
EGOT.1	1.566776708
AC131097.4	1.566668333
AQP12A	1.566307376
GBX2	1.565891065
ALPI	1.563361266
ALPPL2	1.562188556
TM4SF20	1.559155748
AC007879.4	1.559107391
MTND4P23	1.557875529
Y_RNA.125	1.557619721
Clostridiales-1.3	1.557394367
AC092573.2	1.556705224
RP11-20F18.1	1.555924267
AC009961.5	1.555821357
FAM133DP	1.554772343
RPL30P2	1.554596661
NXPH2	1.55421953
RP11-77A13.1	1.554188423
AC017074.3	1.553615743
RP11-1223D19.1	1.552817613
RP11-465O11.2	1.552767464
SULT1C2P1	1.552708254
SULT1C3	1.550813121
SLC5A7	1.549060146
AC092570.1	1.548987808
AC092168.4	1.548385569
AC008268.1	1.544254768

RP11-407P15.2	1.543930383
IGKV1D-16	1.542490501
IGKV1D-39	1.542131961
AC011754.1	1.541691082
REG3A	1.540276986
REG1P	1.539819107
REG1A	1.538980306
REG1B	1.538913047
REG3G	1.538086952
TCEB1P21	1.537520325
CTD-2026C7.1	1.535910004
MIR217HG	1.535224999
AC093732.1	1.533625594
Six3os1_2	1.53354866
RP11-78I14.1	1.533360484
AC073218.3	1.533064781
RP11-674I16.2	1.532520263
AC098828.2	1.532447139
AC017076.1	1.531589995
AC079779.4	1.529940382
RP5-1065P14.2	1.529381698
SNORA51.1	1.529184393
RNA5SP18	1.528476581
RP5-1139B12.4	1.528031424
FAM96AP2	1.527652826
RNU6-403P	1.527056855
Y_RNA.65	1.527042826
RP11-469A15.2	1.526710721
PEBP1P3	1.526655058
RP11-563D10.1	1.526648293
SLC25A38P1	1.525983171
MIR199A2	1.525801774
RP11-506O24.1	1.525768068
RP11-48O20.5	1.525731353
AL359753.1	1.524417662
SNORA80E	1.524373166
MIR555	1.524061475
RP11-439A17.4	1.52108142
HIST2H2BA	1.520989281

HSD3BP4	1.520766717
FTH1P22	1.520111387
RNY1P13	1.519334506
ALX3	1.519262669
AC000032.2	1.518596469
RNU6V	1.517933885
SPATA42	1.517441428
FTLP17	1.516797483
AMY1B	1.516442244
RP11-153F1.1	1.515244079
RP5-1102E8.3	1.51512464
KRT8P21	1.514412478
AL136985.1	1.513972254
RPL21P23	1.513755234
RP11-446E24.3	1.512895243
RP4-758J24.4	1.512356046
RP11-296A18.6	1.512321633
LINC00505	1.512202118
RP11-114B7.6	1.511999188
RP11-266K22.2	1.511456423
uc_338	1.511029567
RN7SL165P	1.510775294
RP11-569G9.7	1.509478839
RP4-654C18.1	1.507647447
RNU6-776P	1.507603977
Metazoa_SRP.6	1.50683794
CELA3A	1.505933179
RNU6-1022P	1.505537505
Metazoa_SRP.5	1.505440616
CELA3B	1.50519848
RP5-1056L3.1	1.505130313
PADI1	1.504794002
RP1-37C10.3	1.504515476
RP11-169K16.4	1.504025571
CELA2B	1.502837099
CELA2A	1.502749605
CTRC	1.502053148
NPPB	1.501716047
MIR6728	1.50147207

RP1-58B11.1	1.500678828
RP11-54O7.10	1.500269826

Supplementary Table S2. Genes whose expression was altered by HOXA11-AS knockdown in KP1-NL cells

Gene name	Fold Change (log2)
ENSG00000273623	9.51193
ENSG00000203812	9.216261
ENSG00000182393	8.175525
ENSG00000276667	8.125104
ENSG00000183709	7.892816
ENSG00000254963	7.8847814
ENSG00000197110	7.508587
ENSG00000256804	7.303872
ENSG00000279225	7.1513715
ENSG00000243104	7.010668
ENSG00000269888	6.931565
ENSG00000121858	6.8685136
ENSG00000279296	6.862132
ENSG00000264391	6.8599696
ENSG00000111581	6.819924
ENSG00000273138	6.814038
ENSG00000267984	6.7869883
ENSG00000277959	6.614857
ENSG00000272558	6.603181
ENSG00000240163	6.5870647
ENSG00000119922	6.521985
ENSG00000252797	6.383359
ENSG00000286901	6.381802
ENSG00000170248	6.3663225
ENSG00000265139	6.3663225
ENSG00000225886	6.3265147
ENSG00000223886	6.274821
ENSG00000177200	6.2431736
ENSG00000181381	6.2214904
ENSG00000132274	6.19928
ENSG00000273813	6.110196
ENSG00000123609	6.0974
ENSG00000243870	6.090007
ENSG00000121905	6.044613
ENSG00000263709	6.0042768
ENSG00000257727	5.999549

ENSG00000089127	5.9314466
ENSG00000273946	5.919101
ENSG00000144228	5.896756
ENSG00000241769	5.870118
ENSG00000274011	5.852248
ENSG00000122729	5.8519835
ENSG00000145016	5.8323836
ENSG00000002549	5.8160877
ENSG00000227081	5.8011584
ENSG00000229601	5.800123
ENSG00000104177	5.7865963
ENSG00000268643	5.771886
ENSG00000276965	5.7626147
ENSG00000267344	5.7279205
ENSG00000285565	5.725196
ENSG00000112667	5.69488
ENSG00000274213	5.6735563
ENSG00000281383	5.6584973
ENSG00000259746	5.635464
ENSG00000122783	5.618142
ENSG00000171311	5.5885644
ENSG00000273132	5.5870647
ENSG00000238151	5.575615
ENSG00000120137	5.528259
ENSG00000234040	5.4937754
ENSG00000263620	5.4825253
ENSG00000142657	5.450661
ENSG00000125730	5.4498916
ENSG00000263558	5.4342947
ENSG00000286874	5.419539
ENSG00000270751	5.408712
ENSG00000248641	5.404971
ENSG00000225864	5.388534
ENSG00000115295	5.3833146
ENSG00000135114	5.3790917
ENSG00000265008	5.3656225
ENSG00000254762	5.3441186
ENSG00000170899	5.3391376
ENSG00000107201	5.329816

ENSG00000237331	5.312883
ENSG00000100342	5.309249
ENSG00000232888	5.3015876
ENSG00000272395	5.28762
ENSG00000285863	5.284292
ENSG00000059378	5.2727695
ENSG00000225822	5.2727695
ENSG00000004799	5.263786
ENSG00000130940	5.2410774
ENSG00000278177	5.23764
ENSG00000276488	5.209453
ENSG00000267198	5.207112
ENSG00000254531	5.2032013
ENSG00000091039	5.19456
ENSG00000156411	5.187451
ENSG00000115267	5.184076
ENSG00000151366	5.1799088
ENSG00000176463	5.177495
ENSG00000014123	5.1767216
ENSG00000243686	5.1751256
ENSG00000154889	5.1647058
ENSG00000247049	5.157852
ENSG00000254400	5.1566396
ENSG00000224401	5.137913
ENSG00000268307	5.1301074
ENSG00000185745	5.129937
ENSG00000244270	5.122673
ENSG00000187608	5.122386
ENSG00000268154	5.110196
ENSG00000026103	5.1001368
ENSG00000185338	5.0950804
ENSG00000230982	5.0925455
ENSG00000180182	5.089583
ENSG00000009413	5.089159
ENSG00000106829	5.089159
ENSG00000248734	5.0849147
ENSG00000128309	5.076379
ENSG00000168807	5.0673804
ENSG00000230224	5.0665197

ENSG00000213903	5.0647964
ENSG00000109689	5.061344
ENSG00000182179	5.0604796
ENSG00000109320	5.0600476
ENSG00000260097	5.059182
ENSG00000270808	5.050066
ENSG00000256262	5.0360637
ENSG00000267135	5.030336
ENSG00000273445	5.0121217
ENSG00000279735	5.0098844
ENSG00000262678	5.0027027
ENSG00000072121	4.9932213
ENSG00000229119	4.9932213
ENSG00000111335	4.9813957
ENSG00000263171	4.9791107
ENSG00000260616	4.9726925
ENSG00000138035	4.957003
ENSG00000243847	4.9527993
ENSG00000198876	4.9429836
ENSG00000163877	4.94188
ENSG00000250264	4.9283705
ENSG00000233271	4.926474
ENSG00000167088	4.9236245
ENSG00000181126	4.9136076
ENSG00000162174	4.8981204
ENSG00000261052	4.8397036
ENSG00000188290	4.753545
ENSG00000157601	4.719639
ENSG00000181027	4.6726346
ENSG00000119917	4.6467857
ENSG00000178685	4.6127014
ENSG00000168062	4.6007457
ENSG00000089902	4.567781
ENSG00000126709	4.554918
ENSG00000197409	4.548063
ENSG00000123124	4.525197
ENSG00000167779	4.4444504
ENSG00000168394	4.431316
ENSG00000173193	4.303159

ENSG00000271503	4.3010416
ENSG00000143294	4.279516
ENSG00000091073	4.256434
ENSG00000074211	4.2411785
ENSG00000114316	4.211553
ENSG00000105939	4.134573
ENSG00000103043	4.130561
ENSG00000140464	4.128599
ENSG00000204261	4.118028
ENSG00000060140	4.101618
ENSG00000082701	4.095967
ENSG00000128335	4.067366
ENSG00000130303	4.0566206
ENSG00000110944	4.0290856
ENSG00000115415	3.9627962
ENSG00000125347	3.9492855
ENSG00000124788	3.9394844
ENSG00000023041	3.9327693
ENSG00000100297	3.8829737
ENSG00000234745	3.8328757
ENSG00000113360	3.8281562
ENSG00000235453	3.8191843
ENSG00000204070	3.8167355
ENSG00000132109	3.7985187
ENSG00000231871	3.790126
ENSG00000121152	3.7770972
ENSG00000141258	3.754499
ENSG00000168781	3.7446837
ENSG00000121644	3.738956
ENSG00000113068	3.7157762
ENSG00000127603	3.7112029
ENSG00000225963	3.6951728
ENSG00000169871	3.6329951
ENSG00000106392	3.6260383
ENSG00000170004	3.6159692
ENSG00000115459	3.601874
ENSG00000229619	3.5871
ENSG00000109606	3.584572
ENSG00000229097	3.5600882

ENSG00000100156	3.5598657
ENSG00000170017	3.5461512
ENSG00000185507	3.5088882
ENSG00000115419	3.503555
ENSG00000116729	3.5029154
ENSG00000214756	3.4867454
ENSG00000271581	3.4830332
ENSG00000130589	3.4714308
ENSG00000265298	3.4702537
ENSG00000257704	3.4679039
ENSG00000184232	3.4531927
ENSG00000101493	3.4339461
ENSG00000234127	3.4167635
ENSG00000197321	3.3862967
ENSG00000112096	3.3736546
ENSG00000137575	3.3735685
ENSG00000155287	3.365988
ENSG00000269680	3.3385026
ENSG00000169246	3.3295147
ENSG00000158156	3.317213
ENSG00000069966	3.2738352
ENSG00000181790	3.258235
ENSG00000105287	3.2470121
ENSG00000151748	3.244575
ENSG00000116133	3.2237475
ENSG00000151376	3.2120655
ENSG00000105559	3.1790562
ENSG00000188313	3.1705477
ENSG00000240771	3.1536987
ENSG00000170581	3.1505475
ENSG00000121680	3.1487048
ENSG00000121060	3.109881
ENSG00000013374	3.108198
ENSG00000111331	3.1031258
ENSG00000273047	3.1018798
ENSG00000072786	3.1007373
ENSG00000053501	3.1001065
ENSG00000174165	3.0815601
ENSG00000150459	3.069461

ENSG00000264982	3.051435
ENSG00000233223	3.046788
ENSG00000228404	3.04256
ENSG00000269915	3.0419762
ENSG00000242086	3.0341601
ENSG00000205362	3.0198627
ENSG00000133835	2.9960794
ENSG00000256116	2.9907699
ENSG00000198959	2.9885907
ENSG00000164609	2.986923
ENSG00000168092	2.982164
ENSG00000101000	2.9820428
ENSG00000110075	2.9689472
ENSG00000079819	2.9666042
ENSG00000100307	2.966098
ENSG00000267387	2.959358
ENSG00000171680	2.9391124
ENSG00000177425	2.9179688
ENSG00000133704	2.9110663
ENSG00000115363	2.908085
ENSG00000153827	2.8971353
ENSG00000221970	2.8907046
ENSG00000183458	2.8869708
ENSG00000108666	2.886038
ENSG00000185880	2.884423
ENSG00000172058	2.878147
ENSG00000092098	2.8735297
ENSG00000119812	2.8639576
ENSG00000213672	2.8625588
ENSG00000100906	2.8536115
ENSG00000125812	2.847867
ENSG00000128342	2.8441548
ENSG00000092330	2.8373497
ENSG00000113161	2.8298366
ENSG00000142082	2.8093052
ENSG00000172465	2.8024287
ENSG00000157873	2.7978575
ENSG00000262112	2.7973647
ENSG00000154803	2.789901

ENSG00000151414	2.782859
ENSG00000171729	2.7789013
ENSG00000008130	2.7709515
ENSG00000166348	2.768568
ENSG00000186063	2.7606013
ENSG00000124201	2.7589023
ENSG00000132256	2.7525356
ENSG00000132819	2.7403765
ENSG00000118873	2.7329135
ENSG00000185803	2.7299192
ENSG00000072274	2.727057
ENSG00000106785	2.724752
ENSG00000100938	2.7234483
ENSG00000116161	2.723085
ENSG00000108588	2.7228158
ENSG00000181788	2.72094
ENSG00000170909	2.720194
ENSG00000078081	2.7082052
ENSG00000095380	2.6928422
ENSG00000228703	2.6863236
ENSG00000260027	2.6855114
ENSG00000254901	2.6811774
ENSG00000167685	2.676272
ENSG00000072849	2.6762614
ENSG00000272115	2.67341
ENSG00000204653	2.6675532
ENSG00000139163	2.667025
ENSG00000129667	2.6659384
ENSG00000168522	2.6486654
ENSG00000060339	2.6405864
ENSG00000172239	2.635986
ENSG00000108679	2.627925
ENSG00000060491	2.6059856
ENSG00000166582	2.6043766
ENSG00000240065	2.5976336
ENSG00000166913	2.5953345
ENSG00000100908	2.5804584
ENSG00000254894	2.5792372
ENSG00000172780	2.5782304

ENSG00000154144	2.5718555
ENSG00000129625	2.5694802
ENSG00000145623	2.5532
ENSG00000232803	2.5513008
ENSG00000177674	2.5478232
ENSG00000139182	2.5350294
ENSG00000129932	2.5340157
ENSG00000168310	2.5213072
ENSG00000157654	2.510859
ENSG00000269893	2.5105844
ENSG00000121057	2.5036626
ENSG00000147471	2.4976463
ENSG00000132842	2.4924529
ENSG00000136240	2.4798303
ENSG00000067840	2.4646993
ENSG00000142089	2.4571352
ENSG00000271821	2.4551277
ENSG00000148824	2.4525843
ENSG00000197982	2.4512844
ENSG00000163512	2.4440928
ENSG00000135899	2.4323988
ENSG00000099308	2.4317558
ENSG00000233825	2.4311678
ENSG00000213753	2.4256494
ENSG00000172183	2.424879
ENSG00000145740	2.424699
ENSG00000067334	2.4232116
ENSG00000164081	2.4203734
ENSG00000124226	2.4084375
ENSG00000099917	2.4051442
ENSG00000130813	2.3993487
ENSG00000133812	2.3925958
ENSG00000145217	2.391522
ENSG00000173726	2.3895926
ENSG00000085365	2.3877454
ENSG00000225610	2.385185
ENSG00000156030	2.3838096
ENSG00000196663	2.3828492
ENSG00000139908	2.381552

ENSG00000277203	2.37866
ENSG00000134744	2.3762734
ENSG00000135423	2.375509
ENSG00000124222	2.3722544
ENSG00000163697	2.3700442
ENSG00000143393	2.3627622
ENSG00000075234	2.3622427
ENSG00000255769	2.359909
ENSG00000169660	2.3523948
ENSG00000105229	2.3498871
ENSG00000275055	2.3411279
ENSG00000075420	2.3376527
ENSG00000225335	2.3342922
ENSG00000227028	2.3329906
ENSG00000228843	2.3244739
ENSG00000197536	2.3179798
ENSG00000187688	2.3142824
ENSG00000186470	2.3072815
ENSG00000159131	2.3032017
ENSG00000075275	2.2777681
ENSG00000100911	2.272705
ENSG00000132207	2.26755
ENSG00000107164	2.2672224
ENSG00000074370	2.2618945
ENSG00000130717	2.2616603
ENSG00000102871	2.2607179
ENSG00000189067	2.2596474
ENSG00000082898	2.2578096
ENSG00000189043	2.252866
ENSG00000255929	2.2478552
ENSG00000235852	2.2387133
ENSG00000117362	2.2359922
ENSG00000115364	2.2351635
ENSG00000181991	2.2252352
ENSG00000037757	2.2229223
ENSG00000138646	2.2183516
ENSG00000170142	2.2163515
ENSG00000101187	2.2144163
ENSG00000204592	2.213521

ENSG00000178028	2.2049801
ENSG00000227091	2.2045903
ENSG00000171159	2.2017984
ENSG00000233558	2.199025
ENSG00000277459	2.1987402
ENSG00000260176	2.195657
ENSG00000261288	2.195657
ENSG00000278367	2.1956518
ENSG00000265967	2.1949177
ENSG00000231970	2.1946778
ENSG00000110011	2.193869
ENSG00000134291	2.1917515
ENSG00000224138	2.1915267
ENSG00000250379	2.1909847
ENSG00000228728	2.188684
ENSG00000007541	2.1857755
ENSG00000212952	2.1831825
ENSG00000162735	2.1804137
ENSG00000025708	2.1757083
ENSG00000187961	2.1739397
ENSG00000166340	2.1738567
ENSG00000267757	2.173451
ENSG00000156587	2.1640294
ENSG00000263069	2.1613379
ENSG00000187186	2.1590953
ENSG00000273443	2.1460161
ENSG00000184557	2.1456075
ENSG00000172057	2.1375039
ENSG00000028528	2.1355436
ENSG00000137100	2.1343505
ENSG00000065802	2.1310744
ENSG00000103942	2.1302938
ENSG00000087510	2.1210523
ENSG00000143382	2.1176074
ENSG00000078140	2.1171236
ENSG00000108821	2.116379
ENSG00000180423	2.1136012
ENSG00000186017	2.1053534
ENSG00000153066	2.1038008

ENSG00000116514	2.1035283
ENSG00000168175	2.101802
ENSG00000069424	2.1013553
ENSG00000124783	2.1005964
ENSG00000148400	2.0974064
ENSG00000117118	2.0969076
ENSG00000229047	2.0954838
ENSG00000161904	2.094405
ENSG00000112297	2.091877
ENSG00000107175	2.0918598
ENSG00000119392	2.0866623
ENSG00000130812	2.082377
ENSG00000184465	2.0818725
ENSG00000114062	2.0805182
ENSG00000067066	2.0758314
ENSG00000197021	2.0756574
ENSG00000278259	2.0641096
ENSG00000109854	2.0614007
ENSG00000204267	2.0604274
ENSG00000213523	2.0494533
ENSG00000104375	2.0490346
ENSG00000268287	2.0452797
ENSG00000141510	2.0426915
ENSG00000130816	2.021138
ENSG00000093144	2.019841
ENSG00000076928	2.017414
ENSG00000100403	2.013824
ENSG00000134825	2.0133672
ENSG00000101104	2.0068831
ENSG00000074181	2.00665
ENSG00000125630	1.9941151
ENSG00000127561	1.9872959
ENSG00000212694	1.9837952
ENSG00000175387	1.9820082
ENSG00000039068	1.9812427
ENSG00000226065	1.9805756
ENSG00000072609	1.9735522
ENSG00000132716	1.9730191
ENSG00000130787	1.9669638

ENSG00000122257	1.957097
ENSG00000174233	1.9508219
ENSG00000115541	1.9423709
ENSG00000068120	1.9404261
ENSG00000132600	1.938504
ENSG00000183495	1.9351695
ENSG00000066117	1.9350505
ENSG00000095319	1.9343579
ENSG00000140740	1.9341774
ENSG00000085644	1.9328864
ENSG00000142549	1.9326315
ENSG00000163463	1.9302173
ENSG00000241370	1.9255912
ENSG00000204209	1.9252362
ENSG00000244045	1.9236081
ENSG00000114993	1.9117675
ENSG00000171953	1.9116879
ENSG00000269910	1.9106855
ENSG00000234678	1.9046426
ENSG00000099899	1.9043827
ENSG00000175215	1.9029717
ENSG00000110844	1.9015255
ENSG00000058673	1.89644
ENSG00000198752	1.8940089
ENSG00000174780	1.8905144
ENSG00000198258	1.8871901
ENSG00000121900	1.8863959
ENSG00000287286	1.8853555
ENSG00000174171	1.8843546
ENSG00000241553	1.8827302
ENSG00000166986	1.8811822
ENSG00000280022	1.8807383
ENSG00000233493	1.8799407
ENSG00000254595	1.8791451
ENSG00000225400	1.8786569
ENSG00000160710	1.8780131
ENSG00000146872	1.8772781
ENSG00000160753	1.8751631
ENSG00000077800	1.8744693

ENSG00000169756	1.8739638
ENSG00000259031	1.8728194
ENSG00000048649	1.8697145
ENSG00000182117	1.8689077
ENSG00000094880	1.866282
ENSG00000130177	1.8646538
ENSG00000260304	1.8633347
ENSG00000242299	1.8633204
ENSG00000141971	1.8630748
ENSG00000160124	1.8577104
ENSG00000158106	1.8563077
ENSG00000123146	1.8534756
ENSG00000173786	1.8512297
ENSG00000110628	1.8468876
ENSG00000163882	1.8455625
ENSG00000143702	1.843869
ENSG00000188157	1.8385954
ENSG00000169220	1.8335755
ENSG00000179943	1.8334043
ENSG00000265519	1.8302388
ENSG00000168040	1.8296607
ENSG00000174521	1.8291936
ENSG00000176531	1.8288093
ENSG00000164054	1.8273387
ENSG00000172845	1.8269742
ENSG00000161847	1.8266337
ENSG00000162337	1.82093
ENSG00000172890	1.8204451
ENSG00000049618	1.8169184
ENSG00000105258	1.8167806
ENSG00000023330	1.8116724
ENSG00000204392	1.8099885
ENSG00000073350	1.8089943
ENSG00000242602	1.8089566
ENSG00000103528	1.8084242
ENSG00000115289	1.8083091
ENSG00000177192	1.80545
ENSG00000204525	1.800745
ENSG00000007255	1.7973797

ENSG00000166289	1.7972703
ENSG00000087111	1.7921691
ENSG00000233231	1.7916632
ENSG00000175768	1.7865162
ENSG00000105443	1.7862606
ENSG00000257529	1.7831593
ENSG00000128928	1.7794836
ENSG00000140332	1.7771611
ENSG00000130741	1.7748859
ENSG00000180573	1.7738309
ENSG00000159202	1.7711811
ENSG00000127125	1.7699144
ENSG00000127554	1.7680955
ENSG00000116254	1.7675114
ENSG00000204388	1.7661929
ENSG00000203896	1.765707
ENSG00000144021	1.7611823
ENSG00000100997	1.7610722
ENSG00000272694	1.7590609
ENSG00000183308	1.7583356
ENSG00000283208	1.755403
ENSG00000255073	1.7552545
ENSG00000198954	1.7545891
ENSG00000105402	1.7512865
ENSG00000187535	1.7486465
ENSG00000146112	1.7463582
ENSG00000064489	1.7337122
ENSG00000065150	1.7326059
ENSG00000123154	1.7296424
ENSG00000131100	1.7289855
ENSG00000255498	1.7282267
ENSG00000280649	1.7216694
ENSG00000132481	1.7213435
ENSG00000128016	1.7129569
ENSG00000181523	1.7055893
ENSG00000135535	1.7035842
ENSG00000272821	1.6981802
ENSG00000232133	1.6974983
ENSG00000136717	1.6947107

ENSG00000204310	1.6934156
ENSG00000177380	1.6920464
ENSG00000160932	1.6916347
ENSG00000196535	1.6911306
ENSG00000183258	1.6901045
ENSG00000129250	1.6892617
ENSG00000125753	1.6875887
ENSG00000227695	1.6858149
ENSG00000242539	1.6793003
ENSG00000185022	1.6760006
ENSG00000176046	1.6739278
ENSG00000197122	1.6695335
ENSG00000161677	1.6672287
ENSG00000146830	1.6657238
ENSG00000236467	1.6611075
ENSG00000187193	1.6598749
ENSG00000273149	1.6562309
ENSG00000250615	1.6541214
ENSG00000171314	1.6461835
ENSG00000188191	1.6394835
ENSG00000124074	1.6387639
ENSG00000282501	1.6353364
ENSG00000132613	1.6340742
ENSG00000154305	1.6326008
ENSG00000139194	1.6312237
ENSG00000156113	1.630799
ENSG00000150687	1.6307287
ENSG00000158195	1.6293466
ENSG00000142606	1.6273904
ENSG00000269335	1.625447
ENSG00000239672	1.6225731
ENSG00000142327	1.6182775
ENSG00000090924	1.6167703
ENSG00000205356	1.6151271
ENSG00000182742	1.6140766
ENSG00000244257	1.6123998
ENSG00000104957	1.6103048
ENSG00000104872	1.6097302
ENSG00000183283	1.6083908

ENSG00000178773	1.6079674
ENSG00000161714	1.6075475
ENSG00000247095	1.604296
ENSG00000093010	1.6019907
ENSG00000112514	1.6015806
ENSG00000278594	1.5974355
ENSG00000229413	1.596746
ENSG00000108256	1.5961056
ENSG00000099622	1.5939455
ENSG00000103490	1.5923777
ENSG00000157778	1.5913923
ENSG00000167986	1.5892277
ENSG00000280893	1.5867026
ENSG00000177606	1.5846229
ENSG00000242265	1.5838044
ENSG00000071127	1.5819983
ENSG00000196510	1.578511
ENSG00000198901	1.5770268
ENSG00000169583	1.5744057
ENSG00000061656	1.5733762
ENSG00000137824	1.5711336
ENSG00000139168	1.5704436
ENSG00000228294	1.565834
ENSG00000109920	1.5650527
ENSG00000253806	1.5631199
ENSG00000197483	1.5608063
ENSG00000123992	1.5580554
ENSG00000119673	1.5574656
ENSG00000237522	1.5517523
ENSG00000120889	1.5511904
ENSG00000141574	1.5463476
ENSG00000169976	1.5425425
ENSG00000103932	1.5411663
ENSG00000089351	1.5318813
ENSG00000107816	1.53123
ENSG00000113384	1.530201
ENSG00000149485	1.5269938
ENSG00000100320	1.5167139
ENSG00000172965	1.515594

ENSG00000275807	1.5114131
ENSG00000172534	1.5109744
ENSG00000204264	1.5069969
ENSG00000103326	1.505693
ENSG00000162613	1.5053864
ENSG00000072958	1.5041032
ENSG00000092010	1.5035143
ENSG00000183726	1.5006661
ENSG00000138430	1.4989448
ENSG00000134287	1.4940705
ENSG00000119655	1.491427
ENSG00000136045	1.4899659
ENSG00000179051	1.4878917
ENSG00000213593	1.4878864
ENSG00000276710	1.4857264
ENSG00000177963	1.4836335
ENSG00000100226	1.4815683
ENSG00000088682	1.4804988
ENSG00000125652	1.4795399
ENSG00000175582	1.475894
ENSG00000163541	1.4749272
ENSG00000131094	1.4748597
ENSG00000185522	1.4712248
ENSG00000181817	1.4685035
ENSG00000126768	1.4617791
ENSG00000271670	1.4610968
ENSG00000137815	1.4608457
ENSG00000183617	1.4607973
ENSG00000100379	1.4592814
ENSG00000122203	1.4574585
ENSG00000206503	1.4564972
ENSG00000277688	1.4541826
ENSG00000116809	1.4540997
ENSG00000185262	1.4536371
ENSG00000119333	1.4519882
ENSG00000178927	1.4509492
ENSG00000100138	1.4501705
ENSG00000087266	1.4500289
ENSG00000130731	1.4486613

ENSG00000233547	1.4479609
ENSG00000122218	1.4466903
ENSG00000163466	1.446322
ENSG00000101266	1.4448695
ENSG00000204120	1.4446254
ENSG00000008394	1.4438255
ENSG00000260350	1.4429932
ENSG00000096746	1.4421024
ENSG00000171208	1.437921
ENSG00000145335	1.4371114
ENSG00000161021	1.435761
ENSG00000010256	1.435493
ENSG00000255320	1.4351587
ENSG00000079432	1.4351053
ENSG00000234338	1.4348946
ENSG00000159228	1.4313855
ENSG00000133606	1.4298582
ENSG00000133027	1.4280269
ENSG00000101084	1.4273796
ENSG00000174891	1.4250441
ENSG00000220583	1.4232259
ENSG00000166710	1.4230938
ENSG00000189159	1.4224133
ENSG00000167772	1.4204607
ENSG00000158552	1.4141884
ENSG00000105643	1.414171
ENSG00000135722	1.410768
ENSG00000197930	1.4101906
ENSG00000131591	1.4098992
ENSG00000126351	1.4094453
ENSG00000030582	1.4078245
ENSG00000277791	1.4046578
ENSG00000147454	1.4037995
ENSG00000269352	1.4026699
ENSG00000185101	1.4000871
ENSG00000057019	1.3990333
ENSG00000255468	1.3938084
ENSG00000070444	1.3906379
ENSG00000173327	1.3882046

ENSG00000282300	1.38379
ENSG00000109501	1.3807325
ENSG00000136193	1.3779488
ENSG00000174021	1.3775973
ENSG00000265749	1.3774266
ENSG00000170525	1.37732
ENSG00000270110	1.3731196
ENSG00000146063	1.3728971
ENSG00000178718	1.371952
ENSG00000180900	1.3693542
ENSG00000064545	1.3685184
ENSG00000184524	1.3676901
ENSG00000260367	1.3674173
ENSG00000141012	1.3671088
ENSG00000039650	1.3655877
ENSG00000100979	1.364126
ENSG00000153179	1.3637359
ENSG00000183020	1.3636227
ENSG00000100242	1.3630419
ENSG00000075089	1.3626945
ENSG00000205339	1.3623137
ENSG00000014919	1.3618743
ENSG00000204220	1.3583074
ENSG00000116649	1.3574181
ENSG00000110057	1.3573298
ENSG00000161202	1.3570695
ENSG00000258663	1.3566637
ENSG00000216490	1.3556008
ENSG00000259955	1.3545818
ENSG00000099326	1.3537333
ENSG00000125398	1.3502159
ENSG00000123143	1.3495679
ENSG00000173442	1.3495555
ENSG00000103023	1.3491149
ENSG00000161204	1.3478544
ENSG00000160410	1.3454809
ENSG00000180879	1.3451653
ENSG00000160888	1.3439355
ENSG00000104980	1.3433275

ENSG00000244693	1.3400211
ENSG00000270792	1.3374085
ENSG00000117859	1.3365726
ENSG00000125148	1.3360329
ENSG00000111231	1.3336391
ENSG00000100321	1.3328722
ENSG00000112759	1.322113
ENSG00000170917	1.3204408
ENSG00000232973	1.3188949
ENSG00000153395	1.3182425
ENSG00000168137	1.3179092
ENSG00000131876	1.3171868
ENSG00000288656	1.3165231
ENSG00000261221	1.316072
ENSG00000099282	1.315805
ENSG00000180228	1.3153167
ENSG00000273611	1.3105507
ENSG00000236383	1.307632
ENSG00000063660	1.3031807
ENSG00000137776	1.3030033
ENSG00000149599	1.3027468
ENSG00000213222	1.3014379
ENSG00000148229	1.2996261
ENSG00000135476	1.2984507
ENSG00000120885	1.2977548
ENSG00000143384	1.2977047
ENSG00000213839	1.2951813
ENSG00000175221	1.2943401
ENSG00000164897	1.2925692
ENSG00000006125	1.2909012
ENSG00000124570	1.2893825
ENSG00000105983	1.289154
ENSG00000149091	1.2873507
ENSG00000198517	1.2866254
ENSG00000101017	1.2842014
ENSG00000111142	1.282598
ENSG00000261054	1.2791243
ENSG00000273599	1.2786171
ENSG00000005486	1.2775593

ENSG00000131788	1.2739224
ENSG00000260111	1.2734661
ENSG00000231999	1.2723827
ENSG00000013364	1.2688842
ENSG00000237278	1.2686467
ENSG00000163702	1.2676063
ENSG00000214944	1.2672422
ENSG00000166734	1.2634516
ENSG00000267598	1.2624121
ENSG00000133226	1.2614317
ENSG00000161642	1.259316
ENSG00000162510	1.2590508
ENSG00000169718	1.257956
ENSG00000279196	1.2577558
ENSG00000174231	1.2545695
ENSG00000265678	1.251234
ENSG00000251151	1.250236
ENSG00000089327	1.2500448
ENSG00000103034	1.2486324
ENSG00000108349	1.2453699
ENSG00000196656	1.2440548
ENSG00000112655	1.2440481
ENSG00000143549	1.2428689
ENSG00000163516	1.241673
ENSG00000131495	1.2379665
ENSG00000110713	1.2356911
ENSG00000185596	1.2344317
ENSG00000131188	1.2338915
ENSG00000169180	1.2307563
ENSG00000121410	1.2300701
ENSG00000085872	1.2287669
ENSG00000182108	1.2271438
ENSG00000120451	1.2241144
ENSG00000167978	1.2214222
ENSG00000239552	1.2205696
ENSG00000197948	1.2194958
ENSG00000147649	1.2185681
ENSG00000102878	1.2181382
ENSG00000131462	1.2156243

ENSG00000003509	1.2135663
ENSG00000147872	1.2131257
ENSG00000117614	1.2128258
ENSG00000251867	1.2128036
ENSG00000174243	1.2103524
ENSG00000111011	1.2086148
ENSG00000111325	1.2052908
ENSG00000111674	1.2042127
ENSG00000134030	1.2027378
ENSG00000106991	1.2023602
ENSG00000164687	1.2009573
ENSG00000142208	1.2004585
ENSG00000062582	1.2004032
ENSG00000050165	1.1970587
ENSG00000280026	1.1949177
ENSG00000267042	1.1943176
ENSG00000266714	1.1929817
ENSG00000069399	1.192452
ENSG00000167595	1.1923771
ENSG00000168273	1.1900339
ENSG00000160408	1.1898007
ENSG00000142687	1.1878867
ENSG00000108557	1.1878088
ENSG00000269482	1.1868334
ENSG00000039560	1.184874
ENSG00000146242	1.1837969
ENSG00000112576	1.1833353
ENSG00000169683	1.1830235
ENSG00000150990	1.181386
ENSG00000131446	1.1806698
ENSG00000169231	1.177062
ENSG00000110696	1.1764016
ENSG00000185129	1.1757653
ENSG00000155363	1.1755719
ENSG00000197912	1.1740217
ENSG00000115091	1.1739631
ENSG00000095066	1.1737223
ENSG00000180891	1.1736488
ENSG00000100425	1.1733665

ENSG00000182934	1.172729
ENSG00000136448	1.1718903
ENSG00000068438	1.17174
ENSG00000285612	1.1710582
ENSG00000175265	1.1701117
ENSG00000125968	1.1695595
ENSG00000172936	1.1667552
ENSG00000286636	1.1662469
ENSG00000211450	1.1634698
ENSG00000133703	1.1622927
ENSG00000175793	1.162066
ENSG00000272267	1.1618481
ENSG00000258985	1.1617503
ENSG00000168894	1.1604867
ENSG00000035862	1.1596875
ENSG00000124177	1.1595111
ENSG00000161547	1.1580458
ENSG00000162512	1.157084
ENSG00000180806	1.1555448
ENSG00000136997	1.1533413
ENSG00000099821	1.1529436
ENSG00000175931	1.1481237
ENSG00000244038	1.1478243
ENSG00000092531	1.1474953
ENSG00000141867	1.1459937
ENSG00000109332	1.1450725
ENSG00000010278	1.1440568
ENSG00000230212	1.1439815
ENSG00000022277	1.1429749
ENSG00000125485	1.14288
ENSG00000105290	1.1418266
ENSG00000163636	1.1416106
ENSG00000141568	1.1389427
ENSG00000266677	1.1386294
ENSG00000068697	1.1370709
ENSG00000135318	1.1365893
ENSG00000129991	1.1364393
ENSG00000270392	1.1362958
ENSG00000148985	1.1358833

ENSG00000197530	1.1348119
ENSG00000267858	1.1339602
ENSG00000119242	1.1331458
ENSG00000100401	1.1320612
ENSG00000145216	1.1268077
ENSG00000138085	1.1260958
ENSG00000126903	1.1244845
ENSG00000197766	1.1223545
ENSG00000101940	1.1212807
ENSG00000043143	1.1179233
ENSG00000106665	1.1166792
ENSG00000160685	1.115952
ENSG00000171223	1.1114235
ENSG00000130758	1.110311
ENSG00000104885	1.1096487
ENSG00000156787	1.1095915
ENSG00000121753	1.1090851
ENSG00000260122	1.1071801
ENSG00000177989	1.1071658
ENSG00000060069	1.1053834
ENSG00000172354	1.1040354
ENSG00000172216	1.101994
ENSG00000263326	1.1015766
ENSG00000225920	1.1010365
ENSG00000116521	1.1006627
ENSG00000270890	1.0990076
ENSG00000166337	1.0982494
ENSG00000100292	1.0975327
ENSG00000214160	1.09723
ENSG00000166333	1.0957994
ENSG00000141562	1.0946679
ENSG00000130304	1.0932832
ENSG00000280435	1.0830641
ENSG00000180398	1.0821424
ENSG00000068308	1.0819983
ENSG00000103254	1.0812316
ENSG00000162910	1.080544
ENSG00000189046	1.0798812
ENSG00000169682	1.0792942

ENSG00000258317	1.0745602
ENSG00000125826	1.0744586
ENSG00000099849	1.0721846
ENSG00000101158	1.072164
ENSG00000127415	1.0709085
ENSG00000168488	1.0698495
ENSG00000164758	1.068223
ENSG00000182272	1.0667887
ENSG00000178127	1.0661602
ENSG00000090447	1.0627699
ENSG00000170779	1.062736
ENSG00000267601	1.0625505
ENSG00000154917	1.0613427
ENSG00000104714	1.060926
ENSG00000132341	1.0600982
ENSG00000168066	1.0584617
ENSG00000173889	1.0574698
ENSG00000142002	1.0573907
ENSG00000131236	1.0566711
ENSG00000168461	1.0538545
ENSG00000213676	1.0526536
ENSG00000268812	1.0526354
ENSG00000004487	1.0496793
ENSG00000123349	1.0481844
ENSG00000155876	1.0476713
ENSG00000277128	1.0476632
ENSG00000232098	1.0473051
ENSG00000183735	1.046123
ENSG00000170854	1.0445786
ENSG00000105321	1.0441313
ENSG00000267064	1.0436225
ENSG00000101294	1.0429955
ENSG00000149823	1.0422006
ENSG00000214300	1.0410485
ENSG00000149577	1.0405917
ENSG00000005206	1.0399256
ENSG00000187720	1.0376287
ENSG00000281490	1.0347447
ENSG00000162729	1.0345969

ENSG00000232358	1.0345488
ENSG00000081307	1.0317602
ENSG00000091542	1.0306907
ENSG00000005882	1.0299911
ENSG00000088832	1.0298505
ENSG00000186019	1.0292454
ENSG00000059573	1.0291634
ENSG00000273891	1.0287046
ENSG00000140545	1.0285792
ENSG00000054282	1.0270324
ENSG00000141524	1.0268044
ENSG00000196954	1.0263629
ENSG00000197070	1.0254483
ENSG00000163249	1.0230484
ENSG00000103145	1.0215259
ENSG00000227212	1.0209107
ENSG00000148248	1.0208569
ENSG00000227640	1.019078
ENSG00000105808	1.0187726
ENSG00000181222	1.0177937
ENSG00000227766	1.016737
ENSG00000165886	1.0163598
ENSG00000125835	1.0156574
ENSG00000164896	1.0147038
ENSG00000143774	1.0133724
ENSG00000159496	1.0126901
ENSG00000172500	1.0122681
ENSG00000095539	1.0093589
ENSG00000132507	1.0076485
ENSG00000250133	1.0070753
ENSG00000272638	1.0064816
ENSG00000113558	1.0062089
ENSG00000256747	1.0045595
ENSG00000120690	1.0040417
ENSG00000186298	1.0034928
ENSG00000249435	1.0032902
ENSG00000119669	1.0013723
ENSG00000166783	0.99911785
ENSG00000145555	0.9989681

ENSG00000177479	0.9974046
ENSG00000164032	0.99632215
ENSG00000130396	0.99557495
ENSG00000158864	0.9955106
ENSG00000135047	0.99478865
ENSG00000110092	0.99373055
ENSG00000246859	0.9934516
ENSG00000126214	0.99298525
ENSG00000188042	0.99289274
ENSG00000105197	0.9927616
ENSG00000160741	0.99254036
ENSG00000100036	0.9922395
ENSG00000140521	0.99210453
ENSG00000203950	0.99125147
ENSG00000266028	0.9898343
ENSG00000251196	0.98797035
ENSG00000235027	0.9875231
ENSG00000120738	0.9871321
ENSG00000043355	0.987123
ENSG00000185033	0.9862976
ENSG00000090339	0.98432064
ENSG00000236083	0.9842987
ENSG00000105669	0.9827914
ENSG00000024422	0.9815488
ENSG00000011304	0.98069906
ENSG00000273419	0.97982216
ENSG00000184432	0.9795418
ENSG00000063180	0.9793148
ENSG00000140443	0.9790735
ENSG00000106397	0.9779234
ENSG00000128564	0.9779086
ENSG00000099864	0.97788715
ENSG00000213024	0.97769356
ENSG00000253438	0.9774318
ENSG00000221869	0.9753747
ENSG00000259780	0.9751868
ENSG00000278603	0.9750252
ENSG00000110200	0.9749613
ENSG00000160209	0.9727087

ENSG00000142409	0.97128963
ENSG00000269858	0.9708185
ENSG00000172732	0.9693332
ENSG00000148335	0.96753645
ENSG00000105426	0.96704817
ENSG00000253645	0.96543455
ENSG00000168724	0.9654174
ENSG00000013563	0.96429586
ENSG00000130764	0.96384954
ENSG00000185049	0.9633384
ENSG00000232295	0.96328497
ENSG00000269145	0.9631386
ENSG00000165526	0.961432
ENSG00000285979	0.960516
ENSG00000116685	0.9591985
ENSG00000169908	0.9585328
ENSG00000184014	0.9581051
ENSG00000280385	0.95769405
ENSG00000213983	0.95728207
ENSG00000117318	0.955997
ENSG00000279047	0.95551395
ENSG00000128050	0.95413923
ENSG00000005448	0.95360136
ENSG00000100811	0.9530027
ENSG00000085788	0.9507508
ENSG00000176340	0.9505892
ENSG00000127948	0.9489145
ENSG00000133316	0.94840336
ENSG00000246451	0.94828606
ENSG00000072210	0.9481206
ENSG00000132522	0.9464164
ENSG00000197903	0.94552755
ENSG00000104341	0.9428024
ENSG00000244151	0.9427099
ENSG00000205937	0.9411063
ENSG00000134107	0.9393573
ENSG00000127586	0.93869495
ENSG00000267801	0.9374666
ENSG00000254192	0.937201

ENSG00000146648	0.9361639
ENSG00000167244	0.9360614
ENSG00000196961	0.933342
ENSG00000239282	0.9321351
ENSG00000175826	0.9305029
ENSG00000173171	0.9283037
ENSG00000125977	0.9281411
ENSG00000197586	0.9253464
ENSG00000198355	0.9252939
ENSG00000108262	0.92455196
ENSG00000234608	0.92161226
ENSG00000142684	0.9191952
ENSG00000230805	0.91607714
ENSG00000140400	0.91594744
ENSG00000064932	0.91559076
ENSG00000178719	0.9147806
ENSG00000149930	0.91251373
ENSG00000136854	0.91247654
ENSG00000122642	0.91153955
ENSG00000135926	0.9111624
ENSG00000105516	0.91040754
ENSG00000281955	0.90790224
ENSG00000120314	0.90790033
ENSG00000286596	0.9072542
ENSG00000107829	0.90684414
ENSG00000170906	0.90668106
ENSG00000204387	0.9053955
ENSG00000073792	0.9036703
ENSG00000149716	0.90321064
ENSG00000167470	0.9019356
ENSG00000280143	0.9016881
ENSG00000103064	0.9015808
ENSG00000158793	0.90146494
ENSG00000181085	0.9007902
ENSG00000166925	0.900064
ENSG00000256034	0.8995352
ENSG00000244230	0.89850855
ENSG00000131238	0.89759874
ENSG00000204843	0.89692545

ENSG00000125755	0.8958392
ENSG00000184900	0.89489126
ENSG00000087365	0.8941145
ENSG00000178951	0.8939233
ENSG00000168610	0.8926339
ENSG00000161996	0.8925419
ENSG00000173486	0.8914833
ENSG00000231925	0.8914261
ENSG00000245112	0.89128876
ENSG00000142627	0.8898506
ENSG00000051128	0.88982725
ENSG00000205771	0.88948154
ENSG00000092820	0.88937426
ENSG00000286435	0.888535
ENSG00000273521	0.8871007
ENSG00000103496	0.8865242
ENSG00000167004	0.8864031
ENSG00000232828	0.8852
ENSG00000166171	0.88317585
ENSG00000185885	0.88264704
ENSG00000260853	0.8825674
ENSG00000238258	0.88194656
ENSG00000186187	0.8811946
ENSG00000185236	0.8807349
ENSG00000256967	0.88070107
ENSG00000065268	0.8801627
ENSG00000170275	0.8800459
ENSG00000166920	0.8779206
ENSG00000253930	0.87727165
ENSG00000061938	0.8772111
ENSG00000215252	0.8771043
ENSG00000123240	0.8762622
ENSG00000162066	0.8758812
ENSG00000134905	0.87575483
ENSG00000255126	0.8751912
ENSG00000204356	0.87465954
ENSG00000213277	0.8717432
ENSG00000087087	0.870286
ENSG00000011422	0.8688612

ENSG00000260500	0.86829424
ENSG00000125844	0.8679347
ENSG00000204822	0.8677144
ENSG00000176915	0.8670411
ENSG00000172009	0.8666396
ENSG00000152291	0.86584806
ENSG00000101421	0.86440516
ENSG00000184922	0.8637934
ENSG00000171453	0.8630748
ENSG00000090013	0.8629575
ENSG00000146963	0.86239004
ENSG00000160767	0.86234856
ENSG00000225032	0.862164
ENSG00000265690	0.86166096
ENSG00000169714	0.86159325
ENSG00000282798	0.859931
ENSG00000152348	0.85984373
ENSG00000143303	0.8592043
ENSG00000105298	0.85874367
ENSG00000272991	0.85836506
ENSG00000116016	0.85821104
ENSG00000282697	0.85803175
ENSG00000188483	0.85502386
ENSG00000133895	0.854342
ENSG00000105135	0.85419416
ENSG00000161981	0.8535547
ENSG00000105364	0.8517828
ENSG00000178913	0.8515439
ENSG00000282668	0.84973145
ENSG00000173905	0.8480773
ENSG00000170027	0.84683275
ENSG00000137817	0.84652996
ENSG00000184058	0.8458128
ENSG00000161960	0.84512806
ENSG00000112787	0.8447604
ENSG00000141551	0.8435879
ENSG00000120075	0.84230375
ENSG00000267765	0.84204674
ENSG00000078618	0.8419671

ENSG00000135924	0.84141874
ENSG00000073584	0.84138346
ENSG00000108639	0.84092903
ENSG00000138757	0.84062815
ENSG00000107281	0.83975697
ENSG00000171853	0.839705
ENSG00000119772	0.838943
ENSG00000175115	0.8381734
ENSG00000136877	0.83745384
ENSG00000130164	0.83744717
ENSG00000171603	0.83713627
ENSG00000148175	0.8361778
ENSG00000139722	0.8334899
ENSG00000267523	0.8326459
ENSG00000133313	0.8324747
ENSG00000233138	0.8313346
ENSG00000259375	0.83023405
ENSG00000181444	0.8299327
ENSG00000157557	0.8291068
ENSG00000129347	0.8284497
ENSG00000219200	0.828321
ENSG00000205746	0.82701635
ENSG00000100241	0.82695866
ENSG00000107262	0.82566977
ENSG00000117868	0.8254061
ENSG00000250902	0.82526827
ENSG00000104388	0.8235235
ENSG00000167601	0.8224015
ENSG00000108828	0.82211494
ENSG00000105325	0.8220663
ENSG00000124214	0.8220291
ENSG00000145901	0.8209748
ENSG00000108561	0.82061386
ENSG00000103353	0.82039785
ENSG00000224186	0.8203821
ENSG00000106605	0.81992435
ENSG00000197965	0.8189697
ENSG00000155506	0.81854534
ENSG00000141696	0.8183913

ENSG00000204316	0.8180146
ENSG00000105058	0.8174076
ENSG00000117410	0.8169942
ENSG00000122861	0.8169646
ENSG00000266036	0.81674004
ENSG00000104894	0.8165412
ENSG00000105982	0.81562424
ENSG00000136811	0.8152976
ENSG00000135821	0.81320286
ENSG00000265415	0.81198645
ENSG00000116478	0.81145906
ENSG00000188229	0.81073236
ENSG00000070610	0.8105979
ENSG00000261505	0.81040955
ENSG00000147274	0.8089037
ENSG00000072110	0.80782557
ENSG00000117395	0.80687666
ENSG00000146083	0.80546284
ENSG00000110321	0.80535173
ENSG00000138600	0.80526304
ENSG00000197324	0.80510855
ENSG00000168259	0.8040862
ENSG00000162526	0.8038268
ENSG00000173581	0.8012271
ENSG00000105699	0.8011298
ENSG00000137767	0.8004546
ENSG00000112335	0.80002165
ENSG00000287299	0.79950047
ENSG00000173473	0.7991042
ENSG00000106290	0.7987962
ENSG00000110651	0.797935
ENSG00000130529	0.79708385
ENSG00000243449	0.7950311
ENSG00000235169	0.79466677
ENSG00000021762	0.79257536
ENSG00000253954	0.79214287
ENSG00000272841	0.7918606
ENSG00000110719	0.79170895
ENSG00000134597	0.7913494

ENSG00000161638	0.7911906
ENSG00000114779	0.79113245
ENSG00000114554	0.7911315
ENSG00000185825	0.78903246
ENSG00000065427	0.78846836
ENSG00000138674	0.7884326
ENSG00000089820	0.78817654
ENSG00000090863	0.7874937
ENSG00000161048	0.7869706
ENSG00000125746	0.7860093
ENSG00000142798	0.7859707
ENSG00000065057	0.78464127
ENSG00000143569	0.78457975
ENSG00000136156	0.78449345
ENSG00000018408	0.78403425
ENSG00000186480	0.78339005
ENSG00000076108	0.7826395
ENSG00000071537	0.78134775
ENSG00000156150	0.78029823
ENSG00000255152	0.7800398
ENSG00000063854	0.7788615
ENSG00000177030	0.77849865
ENSG00000116560	0.77845716
ENSG00000257337	0.7782707
ENSG00000149115	0.7738824
ENSG00000153113	0.77349186
ENSG00000198563	0.7692523
ENSG00000101608	0.76910496
ENSG00000213614	0.76754284
ENSG00000268292	0.7675352
ENSG00000101986	0.7671199
ENSG00000266993	0.76708317
ENSG00000149257	0.7660794
ENSG00000141380	0.76596785
ENSG00000267278	0.7655196
ENSG00000167930	0.7650933
ENSG00000204685	0.7613263
ENSG00000185187	0.7607846
ENSG00000122483	0.7605386

ENSG00000268592	0.7603426
ENSG00000122068	0.7596736
ENSG00000160959	0.7583885
ENSG00000279989	0.75811195
ENSG00000128011	0.75788593
ENSG00000282556	0.7552228
ENSG00000131149	0.7534213
ENSG00000170430	0.75296545
ENSG00000228960	0.75242376
ENSG00000089775	0.75238514
ENSG00000138119	0.75235605
ENSG00000128951	0.7514119
ENSG00000168906	0.7506404
ENSG00000182378	0.7504506
ENSG00000006282	0.74985504
ENSG00000253729	0.7497568
ENSG00000120708	0.74922895
ENSG00000281941	0.7483864
ENSG00000127616	0.7481637
ENSG00000166886	0.74789095
ENSG00000104969	0.7466717
ENSG00000160752	0.7464671
ENSG00000086061	0.7463474
ENSG00000099860	0.74439764
ENSG00000272273	0.7422123
ENSG00000196576	0.7407932
ENSG00000130600	0.7399459
ENSG00000124795	0.73942757
ENSG00000255949	0.73895645
ENSG00000272114	0.7387171
ENSG00000159377	0.7379179
ENSG00000217165	0.7363024
ENSG00000082153	0.7359953
ENSG00000132382	0.7352886
ENSG00000265618	0.73513365
ENSG00000166949	0.73513126
ENSG00000261359	0.7351103
ENSG00000104805	0.7343993
ENSG00000088256	0.73345184

ENSG00000259768	0.73266697
ENSG00000225968	0.73207426
ENSG00000257038	0.7317858
ENSG00000136888	0.7312436
ENSG00000188677	0.72938776
ENSG00000186812	0.7282853
ENSG00000185340	0.72692156
ENSG00000164924	0.7267437
ENSG00000111666	0.72420835
ENSG00000262539	0.7234168
ENSG00000109046	0.7215214
ENSG00000132879	0.720521
ENSG00000050344	0.7204151
ENSG00000166595	0.7192888
ENSG00000180185	0.7183118
ENSG00000250571	0.7181916
ENSG00000115257	0.7163472
ENSG00000267348	0.7163048
ENSG00000063241	0.7156358
ENSG00000178209	0.7156024
ENSG00000104518	0.71452093
ENSG00000125912	0.7135811
ENSG00000183011	0.7133465
ENSG00000141503	0.7120919
ENSG00000163814	0.71188736
ENSG00000160908	0.71131134
ENSG00000100300	0.710516
ENSG00000277156	0.7102623
ENSG00000248015	0.70921135
ENSG00000187735	0.7085829
ENSG00000105993	0.70813084
ENSG00000174106	0.7069082
ENSG00000167797	0.70685434
ENSG00000131473	0.7065177
ENSG00000267169	0.70649004
ENSG00000052749	0.70648384
ENSG00000105339	0.7060046
ENSG00000105612	0.704093
ENSG00000072506	0.70388365

ENSG00000116260	0.7025361
ENSG00000130175	0.7024751
ENSG00000085449	0.7023964
ENSG00000130202	0.70124006
ENSG00000125952	0.700335
ENSG00000218891	0.7002897
ENSG00000136295	0.6955843
ENSG00000184840	0.69556475
ENSG00000198363	0.6945915
ENSG00000268069	0.69434977
ENSG00000072501	0.69365597
ENSG00000263826	0.6928425
ENSG00000071553	0.6926856
ENSG00000125611	0.6916895
ENSG00000171298	0.6915808
ENSG00000141279	0.6914897
ENSG00000131652	0.69095325
ENSG00000138074	0.6897569
ENSG00000137409	0.687469
ENSG00000140398	0.6874094
ENSG00000180596	0.685709
ENSG00000249474	0.6851392
ENSG00000273361	0.6848817
ENSG00000211451	0.6845226
ENSG00000171552	0.68378735
ENSG00000126603	0.68228436
ENSG00000100429	0.68130636
ENSG00000196498	0.68074036
ENSG00000165527	0.68006754
ENSG00000126062	0.6799364
ENSG00000116670	0.677928
ENSG00000185222	0.6777382
ENSG00000101220	0.6769028
ENSG00000230084	0.6764293
ENSG00000011009	0.6752076
ENSG00000115107	0.6739459
ENSG00000273890	0.6737404
ENSG00000197879	0.67235184
ENSG00000014216	0.6722846

ENSG00000184402	0.6699686
ENSG00000151176	0.66895103
ENSG00000141736	0.66894436
ENSG00000165458	0.66855335
ENSG00000122359	0.66840553
ENSG00000139726	0.66603136
ENSG00000279541	0.6640458
ENSG00000170348	0.6633048
ENSG00000117133	0.6629348
ENSG00000131435	0.6627064
ENSG00000177697	0.6621041
ENSG00000185624	0.6619463
ENSG00000157933	0.6613121
ENSG00000172977	0.6610923
ENSG00000204469	0.6587877
ENSG00000092871	0.6583948
ENSG00000123159	0.65821123
ENSG00000256628	0.6581273
ENSG00000114354	0.65734386
ENSG00000175866	0.656847
ENSG00000100600	0.65683794
ENSG00000106628	0.65550804
ENSG00000233885	0.65490055
ENSG00000137693	0.6527972
ENSG00000120093	0.65228033
ENSG00000163902	0.651752
ENSG00000143537	0.6517496
ENSG00000164405	0.65157604
ENSG00000100142	0.6514859
ENSG00000261093	0.6504655
ENSG00000139644	0.6501651
ENSG00000279399	0.64929295
ENSG00000042493	0.6490207
ENSG00000286067	0.64898586
ENSG00000078902	0.6488862
ENSG00000025770	0.6486187
ENSG00000161011	0.6481266
ENSG00000167005	0.64794016
ENSG00000160256	0.6475773

ENSG00000101972	0.64681053
ENSG00000167085	0.64676714
ENSG00000105254	0.6460252
ENSG00000215183	0.64565706
ENSG00000113387	0.6455803
ENSG00000101337	0.64422846
ENSG00000110851	0.6441045
ENSG00000185245	0.6430559
ENSG00000243150	0.6425915
ENSG00000165280	0.6423898
ENSG00000048140	0.641757
ENSG00000100258	0.641273
ENSG00000168569	0.63933706
ENSG00000071655	0.6388402
ENSG00000152558	0.6385236
ENSG00000028839	0.63819885
ENSG00000111678	0.63809633
ENSG00000197562	0.6355634
ENSG00000153406	0.63535213
ENSG00000145494	0.6352873
ENSG00000138434	0.6338501
ENSG00000160285	0.6338496
ENSG00000166136	0.6336441
ENSG00000173575	0.63307095
ENSG00000050820	0.6330018
ENSG00000119318	0.63276243
ENSG00000178585	0.6321235
ENSG00000111716	0.6315141
ENSG00000070404	0.6311369
ENSG00000111639	0.6299553
ENSG00000273729	0.6297126
ENSG00000094631	0.629621
ENSG00000141934	0.6273284
ENSG00000266074	0.62725544
ENSG00000262528	0.6264281
ENSG00000178531	0.6252203
ENSG00000072071	0.62452364
ENSG00000100129	0.6233897
ENSG00000203804	0.62302494

ENSG00000158042	0.6225529
ENSG00000204580	0.6214247
ENSG00000167680	0.62105274
ENSG00000125779	0.6185627
ENSG00000197102	0.61829853
ENSG00000156966	0.6181388
ENSG00000254402	0.61773634
ENSG00000061936	0.61438465
ENSG00000218226	0.6142163
ENSG00000233101	0.61382675
ENSG00000147123	0.61342335
ENSG00000288012	0.612792
ENSG00000217930	0.61257267
ENSG00000186577	0.61178637
ENSG00000158062	0.6094265
ENSG00000010404	0.6082797
ENSG00000007376	0.60757065
ENSG00000268030	0.6055231
ENSG00000152952	0.60438347
ENSG00000119013	0.60422325
ENSG00000115866	0.6041732
ENSG00000263218	0.6024704
ENSG00000160679	0.60204744
ENSG00000141582	0.60171556
ENSG00000175063	0.6012397
ENSG00000090372	0.6009693
ENSG00000137331	0.60070086
ENSG00000133030	0.59813833
ENSG00000177732	0.5970783
ENSG00000254986	0.5953679
ENSG00000126001	0.5950136
ENSG00000273300	0.5946226
ENSG00000263756	0.5940685
ENSG00000160691	0.5939021
ENSG00000141741	0.5936718
ENSG00000090316	0.59283733
ENSG00000177542	0.59129524
ENSG00000265073	0.5911765
ENSG00000132016	0.59063864

ENSG00000099991	0.59017324
ENSG00000247228	0.58828354
ENSG00000204104	0.587697
ENSG00000136026	0.58742046
ENSG00000076351	0.58570004
ENSG00000176490	-0.58496284
ENSG00000260898	-0.5851784
ENSG00000257453	-0.5857506
ENSG00000245648	-0.5863352
ENSG00000257103	-0.58776665
ENSG00000237773	-0.5879407
ENSG00000141698	-0.58928823
ENSG00000149929	-0.5894356
ENSG00000112378	-0.5896578
ENSG00000067606	-0.5897131
ENSG00000110104	-0.5911722
ENSG00000143543	-0.5912776
ENSG00000157916	-0.5913205
ENSG00000007047	-0.59159756
ENSG00000023572	-0.59273815
ENSG00000138443	-0.59459877
ENSG00000012211	-0.5952716
ENSG00000120437	-0.5953164
ENSG00000188186	-0.5993409
ENSG00000269867	-0.60009146
ENSG00000233927	-0.60177135
ENSG00000170540	-0.60248137
ENSG00000100644	-0.6025672
ENSG00000224660	-0.6044564
ENSG00000258504	-0.60588074
ENSG00000264058	-0.60595655
ENSG00000273188	-0.6062217
ENSG00000165516	-0.60708237
ENSG00000106682	-0.6074896
ENSG00000215270	-0.6087613
ENSG00000107771	-0.6112199
ENSG00000100413	-0.61152697
ENSG00000136807	-0.6117244
ENSG00000185046	-0.61180735

ENSG00000130299	-0.6119213
ENSG00000089597	-0.6127472
ENSG00000247556	-0.61310005
ENSG00000168028	-0.61364317
ENSG00000104419	-0.6159673
ENSG00000286037	-0.6159773
ENSG00000197885	-0.616065
ENSG00000162576	-0.6162629
ENSG00000116337	-0.6167569
ENSG00000106245	-0.61919594
ENSG00000213347	-0.6199732
ENSG00000269621	-0.62057257
ENSG00000177182	-0.6216583
ENSG00000287271	-0.6247716
ENSG00000067113	-0.62494993
ENSG00000086232	-0.62555313
ENSG00000096384	-0.6269283
ENSG00000111057	-0.62724495
ENSG00000172819	-0.6284976
ENSG00000288623	-0.6285806
ENSG00000170485	-0.62973356
ENSG00000227198	-0.63017416
ENSG00000162384	-0.6302891
ENSG00000213339	-0.6328387
ENSG00000272004	-0.63399124
ENSG00000167100	-0.63412476
ENSG00000125691	-0.6341944
ENSG00000183696	-0.6344228
ENSG00000145730	-0.6357784
ENSG00000282222	-0.6361246
ENSG00000261684	-0.6368208
ENSG00000279428	-0.63941526
ENSG00000144029	-0.6408849
ENSG00000119705	-0.64179325
ENSG00000109790	-0.64182997
ENSG00000175334	-0.6418953
ENSG00000161791	-0.6423154
ENSG00000114391	-0.64344215
ENSG00000138092	-0.643558

ENSG00000100865	-0.64511776
ENSG00000102181	-0.6451373
ENSG00000265401	-0.646718
ENSG00000085511	-0.64794207
ENSG00000259357	-0.64807224
ENSG00000140497	-0.648139
ENSG00000134480	-0.64944315
ENSG00000171302	-0.65125513
ENSG00000214517	-0.6514249
ENSG00000156860	-0.6518192
ENSG00000223960	-0.6522074
ENSG00000280064	-0.6525459
ENSG00000165912	-0.65258026
ENSG00000268854	-0.65416145
ENSG00000135452	-0.65478754
ENSG00000068323	-0.6554723
ENSG00000118094	-0.6558304
ENSG00000175166	-0.6562004
ENSG00000146457	-0.6579218
ENSG00000278922	-0.6584301
ENSG00000147164	-0.65963745
ENSG00000204842	-0.6601162
ENSG00000101019	-0.6608939
ENSG00000140319	-0.6623659
ENSG00000143256	-0.66509247
ENSG00000169609	-0.6658788
ENSG00000197747	-0.66604805
ENSG00000143443	-0.67109346
ENSG00000075415	-0.6725087
ENSG00000184203	-0.6728573
ENSG00000022840	-0.67407227
ENSG00000197256	-0.67428017
ENSG00000135148	-0.6754012
ENSG00000004975	-0.67543316
ENSG00000138398	-0.6783242
ENSG00000204237	-0.6783371
ENSG00000177731	-0.679039
ENSG00000105974	-0.67938375
ENSG00000081154	-0.6794567

ENSG00000130811	-0.6811943
ENSG00000259583	-0.6819782
ENSG00000233024	-0.6820326
ENSG00000157020	-0.6822424
ENSG00000179222	-0.6858659
ENSG00000167967	-0.6864729
ENSG00000166971	-0.68743324
ENSG00000246174	-0.690886
ENSG00000267724	-0.6932936
ENSG00000275183	-0.6933675
ENSG00000259185	-0.6935959
ENSG00000102858	-0.6936722
ENSG00000279641	-0.6949239
ENSG00000070614	-0.69508505
ENSG00000112309	-0.69693565
ENSG00000280416	-0.69694996
ENSG00000132763	-0.69712543
ENSG00000164587	-0.6974621
ENSG00000286223	-0.69800663
ENSG00000006712	-0.6984782
ENSG00000130939	-0.69860363
ENSG00000081181	-0.70031214
ENSG00000185485	-0.701118
ENSG00000164346	-0.7049856
ENSG00000234961	-0.70513105
ENSG00000173540	-0.70584345
ENSG00000225339	-0.70653296
ENSG00000118900	-0.70795965
ENSG00000259939	-0.70829153
ENSG00000177548	-0.7085848
ENSG00000105185	-0.70999956
ENSG00000103249	-0.71025944
ENSG00000104218	-0.7104931
ENSG00000182979	-0.711627
ENSG00000166526	-0.71188354
ENSG00000186710	-0.7125888
ENSG00000122966	-0.7147279
ENSG00000105856	-0.7180624
ENSG00000134697	-0.7185149

ENSG00000233937	-0.71888685
ENSG00000100029	-0.71917725
ENSG00000119950	-0.7193227
ENSG00000063046	-0.72147036
ENSG00000271787	-0.72397614
ENSG00000068796	-0.7261114
ENSG00000141759	-0.726439
ENSG00000174851	-0.7280588
ENSG00000150995	-0.7281518
ENSG00000071051	-0.7287655
ENSG00000218537	-0.72878647
ENSG00000059122	-0.73140097
ENSG00000174917	-0.73156595
ENSG00000225080	-0.7328849
ENSG00000028116	-0.7347293
ENSG00000088247	-0.7350707
ENSG00000134308	-0.7351103
ENSG00000111276	-0.7355237
ENSG00000198276	-0.7371068
ENSG00000083312	-0.7387409
ENSG00000084774	-0.74001694
ENSG00000151208	-0.7415509
ENSG00000065518	-0.74388456
ENSG00000054118	-0.7452197
ENSG00000174456	-0.74674606
ENSG00000286360	-0.7498741
ENSG00000247595	-0.7499795
ENSG00000258048	-0.7506108
ENSG00000170296	-0.7515435
ENSG00000126458	-0.75154924
ENSG00000139160	-0.7516899
ENSG00000237836	-0.7521014
ENSG00000263424	-0.7534728
ENSG00000177666	-0.7539449
ENSG00000158863	-0.75486135
ENSG00000131470	-0.7552395
ENSG00000171530	-0.7554245
ENSG00000106244	-0.7563238
ENSG00000262533	-0.7575731

ENSG00000066923	-0.7576256
ENSG00000026559	-0.7593117
ENSG00000170633	-0.76052713
ENSG00000131368	-0.7607217
ENSG00000285830	-0.7613697
ENSG00000123131	-0.76300764
ENSG00000198668	-0.7663021
ENSG00000168140	-0.7679901
ENSG00000166311	-0.7709451
ENSG00000186834	-0.77253294
ENSG00000226800	-0.7733593
ENSG00000214413	-0.7734542
ENSG00000226416	-0.7734904
ENSG00000272767	-0.77426577
ENSG00000157227	-0.77485037
ENSG00000005075	-0.7753296
ENSG00000012822	-0.7753997
ENSG00000224713	-0.7773156
ENSG00000262633	-0.7788458
ENSG00000064763	-0.77896976
ENSG00000105393	-0.7794962
ENSG00000011295	-0.77982664
ENSG00000130204	-0.7816949
ENSG00000242485	-0.7818303
ENSG00000169189	-0.7881861
ENSG00000255224	-0.7897968
ENSG00000142453	-0.79182196
ENSG00000112977	-0.7933965
ENSG00000139625	-0.79353
ENSG00000123353	-0.79422855
ENSG00000103423	-0.7952938
ENSG00000267165	-0.79810715
ENSG00000129562	-0.79926825
ENSG00000250365	-0.80208063
ENSG00000267355	-0.80293083
ENSG00000279033	-0.8032665
ENSG00000260179	-0.80387163
ENSG00000143545	-0.8046603
ENSG00000117009	-0.80515766

ENSG00000238197	-0.8053489
ENSG00000127884	-0.80658054
ENSG00000159479	-0.8072548
ENSG00000233077	-0.80853176
ENSG00000145425	-0.8113513
ENSG00000078369	-0.81230164
ENSG00000117242	-0.8125825
ENSG00000109686	-0.8133898
ENSG00000264943	-0.8137331
ENSG00000100311	-0.8147912
ENSG00000185475	-0.8163943
ENSG00000271980	-0.81811714
ENSG00000286790	-0.8187494
ENSG00000134419	-0.8211603
ENSG00000129158	-0.8213978
ENSG00000117450	-0.8220372
ENSG00000146540	-0.8226309
ENSG00000116857	-0.82339954
ENSG00000120742	-0.8236346
ENSG00000173818	-0.8242307
ENSG00000150593	-0.8253536
ENSG00000173545	-0.82538176
ENSG00000261845	-0.8264656
ENSG00000170889	-0.8267784
ENSG00000285329	-0.8282175
ENSG00000134440	-0.8338456
ENSG00000134297	-0.83620214
ENSG00000106400	-0.83775663
ENSG00000146701	-0.83803654
ENSG00000205531	-0.8381715
ENSG00000070087	-0.83843803
ENSG00000163319	-0.8400569
ENSG00000103018	-0.84010077
ENSG00000141002	-0.8407674
ENSG00000258057	-0.8411417
ENSG00000197694	-0.8417735
ENSG00000248774	-0.84306717
ENSG00000128524	-0.8434386
ENSG00000259536	-0.8449683

ENSG00000166262	-0.84507656
ENSG00000120129	-0.8454399
ENSG00000134333	-0.84786606
ENSG00000275700	-0.8493142
ENSG00000245748	-0.849638
ENSG00000105835	-0.85022306
ENSG00000137880	-0.8508239
ENSG00000174437	-0.8517175
ENSG00000250033	-0.8526864
ENSG00000123119	-0.85279846
ENSG00000279504	-0.8548207
ENSG00000170043	-0.8559971
ENSG00000135090	-0.85682917
ENSG00000259781	-0.8592701
ENSG00000090581	-0.8605571
ENSG00000167969	-0.8613105
ENSG00000106263	-0.8615079
ENSG00000268199	-0.8616967
ENSG00000117139	-0.8625355
ENSG00000271984	-0.86367846
ENSG00000127952	-0.8648405
ENSG00000230698	-0.8651738
ENSG00000272599	-0.8658776
ENSG00000225507	-0.8665581
ENSG00000278769	-0.86702824
ENSG00000277258	-0.8700161
ENSG00000175467	-0.8705225
ENSG00000203469	-0.87302065
ENSG00000137203	-0.8740096
ENSG00000139278	-0.8747802
ENSG00000130640	-0.874908
ENSG00000213186	-0.8754573
ENSG00000138101	-0.8773904
ENSG00000004142	-0.87913275
ENSG00000257270	-0.8808651
ENSG00000181350	-0.8819299
ENSG00000254198	-0.8848767
ENSG00000128185	-0.88559437
ENSG00000273275	-0.8871527

ENSG00000279413	-0.8880763
ENSG00000143977	-0.88837767
ENSG00000241343	-0.88960075
ENSG00000130522	-0.890121
ENSG00000136003	-0.892066
ENSG00000230583	-0.89262676
ENSG00000114054	-0.89275026
ENSG00000287967	-0.8955612
ENSG00000139428	-0.8964839
ENSG00000213619	-0.8985863
ENSG00000272941	-0.9008517
ENSG00000249042	-0.902287
ENSG00000113141	-0.90238094
ENSG00000197785	-0.90260696
ENSG00000105771	-0.90293884
ENSG00000185686	-0.9031744
ENSG00000213923	-0.9043107
ENSG00000183207	-0.90529203
ENSG00000261822	-0.9058614
ENSG00000242261	-0.90707064
ENSG00000176890	-0.9088898
ENSG00000168779	-0.9096627
ENSG00000261063	-0.9108281
ENSG00000189403	-0.9111657
ENSG00000134686	-0.911252
ENSG00000259515	-0.9131775
ENSG00000259049	-0.91345406
ENSG00000227057	-0.9141326
ENSG00000198034	-0.91739655
ENSG00000121579	-0.9185529
ENSG00000167552	-0.918694
ENSG00000142733	-0.91976166
ENSG00000125744	-0.9200406
ENSG00000075151	-0.92087793
ENSG00000006704	-0.9216752
ENSG00000198131	-0.921721
ENSG00000133872	-0.9223542
ENSG00000142168	-0.9230671
ENSG00000107984	-0.92479706

ENSG00000162878	-0.92772675
ENSG00000187147	-0.92975235
ENSG00000260276	-0.9300723
ENSG00000162520	-0.93079615
ENSG00000084754	-0.9312949
ENSG00000228170	-0.9323349
ENSG00000118898	-0.93387127
ENSG00000115685	-0.9344702
ENSG00000197548	-0.9346895
ENSG00000126934	-0.93485117
ENSG00000163754	-0.9350567
ENSG00000164889	-0.93568516
ENSG00000108788	-0.9359937
ENSG00000243762	-0.9370208
ENSG00000270728	-0.9372349
ENSG00000163959	-0.94188595
ENSG00000280320	-0.9420147
ENSG00000170356	-0.943913
ENSG00000137161	-0.94443035
ENSG00000136238	-0.9452009
ENSG00000169228	-0.94902325
ENSG00000130734	-0.95280886
ENSG00000120068	-0.95425034
ENSG00000253492	-0.9558306
ENSG00000254335	-0.9558306
ENSG00000167515	-0.95829105
ENSG00000231955	-0.95874643
ENSG00000244187	-0.96321154
ENSG00000136213	-0.96353245
ENSG00000108604	-0.9676256
ENSG00000182054	-0.9677758
ENSG00000258315	-0.97031116
ENSG00000268309	-0.97151375
ENSG00000251095	-0.97333384
ENSG00000261465	-0.97451496
ENSG00000168924	-0.9775255
ENSG00000108946	-0.9785013
ENSG00000159596	-0.979414
ENSG00000136731	-0.98036146

ENSG00000090061	-0.98309135
ENSG00000068903	-0.9836173
ENSG00000169715	-0.98373604
ENSG00000269792	-0.9847193
ENSG00000162076	-0.98798513
ENSG00000120725	-0.99001455
ENSG00000248671	-0.9901426
ENSG00000240342	-0.9906249
ENSG00000248924	-0.9908719
ENSG00000184047	-0.9912491
ENSG00000179010	-0.9929261
ENSG00000198522	-0.9966135
ENSG00000261790	-1.0018535
ENSG00000196262	-1.0059013
ENSG00000145741	-1.0061622
ENSG00000110880	-1.0064855
ENSG00000136930	-1.0070019
ENSG00000111642	-1.0074043
ENSG00000131037	-1.0097485
ENSG00000187109	-1.0110388
ENSG00000198917	-1.0145793
ENSG00000269427	-1.0149899
ENSG00000090006	-1.0184112
ENSG00000159692	-1.020916
ENSG00000088833	-1.0212545
ENSG00000132780	-1.0237718
ENSG00000196937	-1.0244493
ENSG00000153044	-1.0267935
ENSG00000263585	-1.0281696
ENSG00000165716	-1.0291281
ENSG00000236883	-1.0297899
ENSG00000286431	-1.0306273
ENSG00000058600	-1.0306649
ENSG00000182472	-1.0307798
ENSG00000100380	-1.0310683
ENSG00000092199	-1.0333128
ENSG00000160014	-1.0373902
ENSG00000088448	-1.0378051
ENSG00000232940	-1.0462513

ENSG00000283463	-1.0505404
ENSG00000263843	-1.0513334
ENSG00000236756	-1.0525694
ENSG00000170606	-1.0530877
ENSG00000148908	-1.0535493
ENSG00000198369	-1.0539241
ENSG00000125459	-1.0561762
ENSG00000115318	-1.0563099
ENSG00000139112	-1.0582561
ENSG00000260349	-1.062573
ENSG00000196295	-1.0629153
ENSG00000280071	-1.0631995
ENSG00000136560	-1.0643306
ENSG00000173153	-1.0667238
ENSG00000162521	-1.0669065
ENSG00000107862	-1.0677285
ENSG00000165233	-1.0705714
ENSG00000148296	-1.0758214
ENSG00000106330	-1.0769095
ENSG00000154832	-1.0773778
ENSG00000182149	-1.080792
ENSG00000186468	-1.0808392
ENSG00000112293	-1.082639
ENSG00000062485	-1.0854154
ENSG00000107949	-1.0887609
ENSG00000143924	-1.0899458
ENSG00000125841	-1.0903482
ENSG00000169045	-1.0933194
ENSG00000089289	-1.093986
ENSG00000188687	-1.0950484
ENSG00000142655	-1.09622
ENSG00000135624	-1.0970564
ENSG00000236723	-1.0984554
ENSG00000176623	-1.1029186
ENSG00000128463	-1.1062436
ENSG00000164307	-1.1077399
ENSG00000179564	-1.1104331
ENSG00000138303	-1.1126537
ENSG00000255467	-1.114614

ENSG00000175197	-1.1160274
ENSG00000248881	-1.1184945
ENSG00000171222	-1.1188645
ENSG00000004779	-1.1213489
ENSG00000099866	-1.1233182
ENSG00000132635	-1.1244164
ENSG00000105486	-1.1244254
ENSG00000163170	-1.1262145
ENSG00000184863	-1.1262727
ENSG00000184752	-1.1267948
ENSG00000147813	-1.1273651
ENSG00000250222	-1.1285701
ENSG00000124422	-1.129056
ENSG00000125966	-1.1292276
ENSG00000214401	-1.1299624
ENSG00000166855	-1.1302223
ENSG00000233033	-1.1305156
ENSG00000147604	-1.1314363
ENSG00000106089	-1.1320677
ENSG00000163481	-1.1336722
ENSG00000138079	-1.1347213
ENSG00000215845	-1.1369839
ENSG00000119632	-1.138371
ENSG00000142396	-1.1386452
ENSG00000278977	-1.1392083
ENSG00000113368	-1.1392751
ENSG00000223374	-1.1393733
ENSG00000228021	-1.1394281
ENSG00000267049	-1.1424508
ENSG00000125356	-1.1437283
ENSG00000243989	-1.1447086
ENSG00000108179	-1.1460032
ENSG00000127922	-1.146636
ENSG00000089159	-1.1477666
ENSG00000270820	-1.151649
ENSG00000100109	-1.1520028
ENSG00000257285	-1.1526537
ENSG00000141367	-1.152884
ENSG00000140548	-1.1546712

ENSG00000076554	-1.1560459
ENSG00000079616	-1.159884
ENSG00000213585	-1.1605635
ENSG00000089220	-1.1610532
ENSG00000126705	-1.1659584
ENSG00000273308	-1.168879
ENSG00000279945	-1.1710014
ENSG00000031698	-1.173315
ENSG00000177728	-1.1740937
ENSG00000137831	-1.1778607
ENSG00000109113	-1.1784449
ENSG00000064102	-1.1793635
ENSG00000074657	-1.1794705
ENSG00000145907	-1.1813393
ENSG00000141027	-1.1824903
ENSG00000111237	-1.1869326
ENSG00000132676	-1.1922283
ENSG00000203875	-1.1929188
ENSG00000177889	-1.1958427
ENSG00000213639	-1.196116
ENSG00000272734	-1.196836
ENSG00000116586	-1.1969571
ENSG00000144591	-1.1987593
ENSG00000163608	-1.1995926
ENSG00000229689	-1.200757
ENSG00000170473	-1.201426
ENSG00000065526	-1.2057853
ENSG00000165916	-1.2069421
ENSG00000246334	-1.2072458
ENSG00000090530	-1.2090602
ENSG00000171206	-1.210393
ENSG00000087274	-1.2167869
ENSG00000136404	-1.21735
ENSG00000156976	-1.2207117
ENSG00000131669	-1.2242522
ENSG00000258388	-1.2257872
ENSG00000102931	-1.2265863
ENSG00000197782	-1.229672
ENSG00000267576	-1.2308054

ENSG00000028203	-1.2333229
ENSG00000235652	-1.2342944
ENSG00000215908	-1.2353098
ENSG00000144381	-1.2387261
ENSG00000169223	-1.239181
ENSG00000162231	-1.2394662
ENSG00000100462	-1.2414823
ENSG00000149547	-1.2417696
ENSG00000144567	-1.2442365
ENSG00000185896	-1.246418
ENSG00000226167	-1.2464924
ENSG00000143156	-1.2516427
ENSG00000141522	-1.2539759
ENSG00000132254	-1.2577887
ENSG00000159079	-1.2578387
ENSG00000236498	-1.2593746
ENSG00000117399	-1.261162
ENSG00000140992	-1.2644053
ENSG00000111667	-1.2647445
ENSG00000077312	-1.2672696
ENSG00000059145	-1.2674923
ENSG00000267458	-1.2678089
ENSG00000131467	-1.2678328
ENSG00000263986	-1.2691255
ENSG00000142856	-1.2697701
ENSG00000288066	-1.2711945
ENSG00000126870	-1.2755017
ENSG00000114395	-1.2755809
ENSG00000258377	-1.2764282
ENSG00000135446	-1.2764645
ENSG00000083857	-1.2813058
ENSG00000111652	-1.284024
ENSG00000140632	-1.2848587
ENSG00000066230	-1.2874908
ENSG00000117676	-1.2882872
ENSG00000178252	-1.2887807
ENSG00000123106	-1.2909555
ENSG00000224272	-1.2972226
ENSG00000079246	-1.2974381

ENSG00000275185	-1.2981625
ENSG00000172115	-1.298279
ENSG00000277801	-1.2989268
ENSG00000183718	-1.3042431
ENSG00000131748	-1.3086231
ENSG00000129353	-1.3106203
ENSG00000102317	-1.31215
ENSG00000164104	-1.3126998
ENSG00000180817	-1.3162723
ENSG00000144224	-1.3211374
ENSG00000130479	-1.3214669
ENSG00000187531	-1.3233047
ENSG00000091640	-1.3235207
ENSG00000148826	-1.3261962
ENSG00000113732	-1.3333945
ENSG00000105677	-1.3366961
ENSG00000236552	-1.3400075
ENSG00000231822	-1.3419976
ENSG00000245532	-1.3430338
ENSG00000100554	-1.3442178
ENSG00000022267	-1.3474379
ENSG00000144659	-1.3490033
ENSG00000143436	-1.3498354
ENSG00000105245	-1.3512683
ENSG00000197006	-1.3605475
ENSG00000172893	-1.3739223
ENSG00000197457	-1.3743458
ENSG00000115414	-1.3751101
ENSG00000169972	-1.3766184
ENSG00000060971	-1.3794436
ENSG00000177383	-1.3823357
ENSG00000162923	-1.3828311
ENSG00000229036	-1.3833752
ENSG00000109805	-1.3840313
ENSG00000114126	-1.3867595
ENSG00000134684	-1.3874183
ENSG00000260549	-1.3881807
ENSG00000003756	-1.3887944
ENSG00000196497	-1.391393

ENSG00000141646	-1.3922186
ENSG00000130244	-1.3930931
ENSG00000135390	-1.3954539
ENSG00000176155	-1.4070764
ENSG00000006607	-1.4096129
ENSG00000090861	-1.4105191
ENSG00000010292	-1.413167
ENSG00000231864	-1.4181676
ENSG00000263276	-1.4200447
ENSG00000269972	-1.4214029
ENSG00000113621	-1.4228678
ENSG00000008838	-1.4252105
ENSG00000100296	-1.4269905
ENSG00000163683	-1.427238
ENSG00000204394	-1.436533
ENSG00000117592	-1.4381652
ENSG00000079332	-1.4397957
ENSG00000106976	-1.4472089
ENSG00000214889	-1.4477987
ENSG00000272842	-1.4487877
ENSG00000254756	-1.4519176
ENSG00000169398	-1.4570556
ENSG00000163166	-1.4580734
ENSG00000029993	-1.4621129
ENSG00000257553	-1.4629936
ENSG00000122545	-1.4640388
ENSG00000104131	-1.4677896
ENSG00000150403	-1.4681716
ENSG00000273261	-1.4681745
ENSG00000132680	-1.4682999
ENSG00000167964	-1.4683013
ENSG00000169567	-1.4710474
ENSG00000155368	-1.471118
ENSG00000165502	-1.4785328
ENSG00000176476	-1.4840374
ENSG00000113328	-1.489212
ENSG00000126012	-1.4895515
ENSG00000008952	-1.5006433
ENSG00000123815	-1.5059557

ENSG00000277494	-1.5066631
ENSG00000127837	-1.5117393
ENSG00000133250	-1.5172429
ENSG00000100664	-1.5183959
ENSG00000109339	-1.5230553
ENSG00000136938	-1.5366597
ENSG00000273485	-1.5368683
ENSG00000130810	-1.5395675
ENSG00000226803	-1.5429983
ENSG00000265241	-1.5444593
ENSG00000168036	-1.5452547
ENSG00000178057	-1.5484157
ENSG00000272196	-1.5501089
ENSG00000114439	-1.55229
ENSG00000125447	-1.5559368
ENSG00000168214	-1.5565755
ENSG00000039319	-1.5716138
ENSG00000100266	-1.5761209
ENSG00000174547	-1.5803404
ENSG00000174775	-1.5821018
ENSG00000149792	-1.5951436
ENSG00000243716	-1.5955329
ENSG00000110921	-1.6088321
ENSG00000221968	-1.623877
ENSG00000154640	-1.6247218
ENSG00000275857	-1.6292481
ENSG00000110801	-1.6296892
ENSG00000272345	-1.6299877
ENSG00000156467	-1.6333652
ENSG00000167513	-1.6347358
ENSG00000286070	-1.6508064
ENSG00000040275	-1.6514144
ENSG00000157045	-1.6563058
ENSG00000100949	-1.6575811
ENSG00000265778	-1.6589952
ENSG00000131584	-1.6696725
ENSG00000243509	-1.6746864
ENSG00000168872	-1.68012
ENSG00000214736	-1.6836102

ENSG00000104774	-1.6888971
ENSG00000248932	-1.6911457
ENSG00000229809	-1.6960409
ENSG00000253854	-1.7000449
ENSG00000166173	-1.7002969
ENSG00000112578	-1.7003131
ENSG00000149177	-1.7045791
ENSG00000168653	-1.7072988
ENSG00000178188	-1.7090878
ENSG00000267232	-1.7106338
ENSG00000182173	-1.7106633
ENSG00000268191	-1.7151613
ENSG00000228275	-1.7178342
ENSG00000140382	-1.7258253
ENSG00000215154	-1.7264998
ENSG00000227278	-1.7266183
ENSG00000132432	-1.7275944
ENSG00000125944	-1.72897
ENSG00000259238	-1.7335024
ENSG00000265168	-1.7337599
ENSG00000005059	-1.7341523
ENSG00000174446	-1.7375488
ENSG00000141198	-1.7376902
ENSG00000184162	-1.7387908
ENSG00000231084	-1.7416723
ENSG00000267448	-1.7424324
ENSG00000064607	-1.7515631
ENSG00000188846	-1.7541428
ENSG00000164062	-1.7548103
ENSG00000132768	-1.7600384
ENSG00000287496	-1.7748122
ENSG00000151348	-1.778981
ENSG00000184154	-1.7800908
ENSG00000112146	-1.7849827
ENSG00000136819	-1.7857792
ENSG00000241114	-1.7876897
ENSG00000140455	-1.7944672
ENSG00000169439	-1.8115954
ENSG00000078142	-1.824475

ENSG00000167257	-1.8343873
ENSG00000161217	-1.8346856
ENSG00000124006	-1.8386211
ENSG00000109685	-1.8395648
ENSG00000177565	-1.8444116
ENSG00000089009	-1.8605223
ENSG00000080824	-1.8676133
ENSG00000137171	-1.8728724
ENSG00000163806	-1.87503
ENSG00000228509	-1.8799489
ENSG00000123444	-1.8823533
ENSG00000168827	-1.8851454
ENSG00000005187	-1.896081
ENSG00000136574	-1.9130237
ENSG00000255284	-1.9165463
ENSG00000214530	-1.9175873
ENSG00000261207	-1.9335473
ENSG00000269813	-1.9365897
ENSG00000114796	-1.9403303
ENSG00000115657	-1.9449272
ENSG00000135372	-1.964581
ENSG00000283375	-1.9652452
ENSG00000144895	-1.9661682
ENSG00000261113	-1.970756
ENSG00000224645	-1.9713459
ENSG00000150779	-1.9771934
ENSG00000272391	-1.9900901
ENSG00000206140	-1.9948184
ENSG00000129680	-1.9950261
ENSG00000173915	-2.0105412
ENSG00000165804	-2.0128484
ENSG00000163930	-2.0152397
ENSG00000198431	-2.029029
ENSG00000168517	-2.0411797
ENSG00000037241	-2.0422368
ENSG00000280400	-2.0430937
ENSG00000104522	-2.0440147
ENSG00000196465	-2.0503263
ENSG00000129993	-2.052809

ENSG00000109917	-2.0546992
ENSG00000089006	-2.0621254
ENSG00000117616	-2.0656574
ENSG00000153774	-2.072751
ENSG00000204991	-2.0776253
ENSG00000130311	-2.0816836
ENSG00000286264	-2.0868843
ENSG00000143314	-2.0891304
ENSG00000231770	-2.1059365
ENSG00000106636	-2.1106563
ENSG00000265188	-2.1151729
ENSG00000166012	-2.115182
ENSG00000251675	-2.121736
ENSG00000225108	-2.1256614
ENSG00000283703	-2.1266422
ENSG00000253445	-2.1293755
ENSG00000268496	-2.1334407
ENSG00000258515	-2.1341717
ENSG00000002822	-2.136643
ENSG00000087076	-2.1484847
ENSG00000131242	-2.1580718
ENSG00000105429	-2.1607296
ENSG00000115042	-2.1778712
ENSG00000090674	-2.1807942
ENSG00000286623	-2.1816201
ENSG00000183684	-2.1828418
ENSG00000198837	-2.1984034
ENSG00000111540	-2.205514
ENSG00000087253	-2.23025
ENSG00000267073	-2.2442155
ENSG00000182446	-2.2774177
ENSG00000284969	-2.2853642
ENSG00000223343	-2.2879856
ENSG00000267580	-2.2915475
ENSG00000272087	-2.2922566
ENSG00000166226	-2.2931895
ENSG00000113643	-2.29649
ENSG00000015676	-2.2994392
ENSG00000288065	-2.3355405

ENSG00000101460	-2.3432026
ENSG00000151883	-2.359024
ENSG00000215375	-2.3612082
ENSG00000267470	-2.3645875
ENSG00000197989	-2.3683655
ENSG00000148834	-2.368947
ENSG00000204536	-2.3800075
ENSG00000267062	-2.3871512
ENSG00000137501	-2.404854
ENSG00000177303	-2.4151661
ENSG00000113407	-2.4237242
ENSG00000106258	-2.435386
ENSG00000266261	-2.4486482
ENSG00000243305	-2.4573536
ENSG00000273369	-2.4614987
ENSG00000253210	-2.4947646
ENSG00000235174	-2.502244
ENSG00000279457	-2.5042124
ENSG00000198000	-2.5136335
ENSG00000197172	-2.5154483
ENSG00000081692	-2.518042
ENSG00000259792	-2.5263124
ENSG00000173531	-2.5300076
ENSG00000011426	-2.5594952
ENSG00000131174	-2.5736914
ENSG00000167283	-2.6020787
ENSG00000106012	-2.6096907
ENSG00000217801	-2.6121027
ENSG00000131871	-2.6148827
ENSG00000008256	-2.6331208
ENSG00000273154	-2.643965
ENSG00000165914	-2.6457276
ENSG00000198792	-2.6566947
ENSG00000182903	-2.6633544
ENSG00000205078	-2.6770139
ENSG00000120896	-2.6900876
ENSG00000198826	-2.706149
ENSG00000238186	-2.7128341
ENSG00000167186	-2.715655

ENSG00000254680	-2.7171798
ENSG00000167535	-2.7199168
ENSG00000254578	-2.727313
ENSG00000198865	-2.7312305
ENSG00000131095	-2.7485538
ENSG00000242612	-2.753262
ENSG00000197345	-2.7534099
ENSG00000100599	-2.768474
ENSG00000086666	-2.7724657
ENSG00000254814	-2.8182185
ENSG00000113658	-2.8544855
ENSG00000108883	-2.8687918
ENSG00000156502	-2.86988
ENSG00000220793	-2.9080594
ENSG00000002330	-2.9583526
ENSG00000280734	-2.9688776
ENSG00000167695	-2.9784582
ENSG00000163975	-2.9846792
ENSG00000108578	-2.9868028
ENSG00000274859	-2.9901342
ENSG00000091136	-2.9957957
ENSG00000223768	-3.0156882
ENSG00000108443	-3.0501897
ENSG00000155508	-3.0561798
ENSG00000155957	-3.0644586
ENSG00000124275	-3.0684009
ENSG00000138768	-3.0805526
ENSG00000082068	-3.1477327
ENSG00000106541	-3.2240372
ENSG00000101144	-3.23951
ENSG00000100124	-3.242586
ENSG00000034713	-3.2684631
ENSG00000054116	-3.2695184
ENSG00000253716	-3.2927818
ENSG00000075303	-3.3593636
ENSG00000251192	-3.4124246
ENSG00000220205	-3.4230843
ENSG00000156603	-3.4820623
ENSG00000267575	-3.5445735

ENSG00000146729	-3.566729
ENSG00000225393	-3.5872915
ENSG00000233429	-3.6364326
ENSG00000103005	-3.6503603
ENSG00000049541	-3.665666
ENSG00000118515	-3.6960123
ENSG00000234664	-3.71984
ENSG00000226312	-3.7243657
ENSG00000164978	-3.7345853
ENSG00000132849	-3.7807608
ENSG00000069275	-3.8173933
ENSG00000130822	-3.8716264
ENSG00000075239	-3.9279995
ENSG00000166946	-3.954196
ENSG00000265511	-4.024316
ENSG00000205089	-4.054967
ENSG00000268565	-4.057684
ENSG00000139323	-4.087848
ENSG00000087338	-4.3100953
ENSG00000075292	-4.4238453
ENSG00000137210	-4.4306197
ENSG00000118363	-4.5026026
ENSG00000266338	-4.515507
ENSG00000235162	-4.62555
ENSG00000133315	-4.625744
ENSG00000163257	-4.6671886
ENSG00000196597	-4.9073715
ENSG00000273160	-4.943921
ENSG00000043093	-4.954196
ENSG00000242207	-5.0214796
ENSG00000164902	-5.0400157
ENSG00000243339	-5.0574503
ENSG00000181938	-5.0669503
ENSG00000271889	-5.0682406
ENSG00000273183	-5.080658
ENSG00000010219	-5.08151
ENSG00000143815	-5.0849147
ENSG00000163634	-5.087887
ENSG00000259187	-5.099295

ENSG00000268729	-5.1251554
ENSG00000147905	-5.146492
ENSG00000288258	-5.185074
ENSG00000123415	-5.193772
ENSG00000168701	-5.199672
ENSG00000188710	-5.231125
ENSG00000260618	-5.232277
ENSG00000267059	-5.2341948
ENSG00000228532	-5.240696
ENSG00000112852	-5.244126
ENSG00000254527	-5.246788
ENSG00000226143	-5.249066
ENSG00000258092	-5.261907
ENSG00000143429	-5.2993913
ENSG00000144785	-5.3179555
ENSG00000127774	-5.3504972
ENSG00000224195	-5.3610663
ENSG00000230325	-5.377818
ENSG00000267796	-5.380591
ENSG00000260639	-5.388534
ENSG00000272523	-5.424922
ENSG00000258283	-5.439955
ENSG00000271532	-5.439955
ENSG00000271973	-5.439955
ENSG00000129460	-5.446587
ENSG00000163539	-5.451541
ENSG00000272836	-5.470537
ENSG00000228802	-5.476382
ENSG00000279226	-5.545351
ENSG00000272600	-5.556123
ENSG00000213413	-5.563158
ENSG00000227053	-5.5665107
ENSG00000259409	-5.5762196
ENSG00000214140	-5.5858636
ENSG00000227409	-5.5891643
ENSG00000123009	-5.5936522
ENSG00000079257	-5.6049604
ENSG00000261064	-5.6311045
ENSG00000244692	-5.631686

ENSG00000275506	-5.6758156
ENSG00000237259	-5.9752173
ENSG00000239856	-6.0382605
ENSG00000179085	-6.0695305
ENSG00000243562	-6.096978
ENSG00000228415	-6.160275
ENSG00000235802	-6.232853
ENSG00000265794	-6.336819
ENSG00000259169	-6.355439
ENSG00000234271	-6.3692923
ENSG00000277978	-6.4646683
ENSG00000221496	-6.551362
ENSG00000183648	-6.9055676
ENSG00000224543	-7.146492
ENSG00000277922	-7.297375
ENSG00000233838	-8.2367325
ENSG00000272478	-8.722091
ENSG00000173727	-9.278357

Supplementary Table S3. Sequences of the siRNAs used in this study

Name	Sequence
Control DsiRNA	NA (Cat#51-01-14-04)
si-HOXA11-AS-1	5'-AAGGAGAUUUGGUCAGCAAAACAGA-3'
si-HOXA11-AS-2	5'-GGUGACUUGAUUACACUCUCUCATT-3'
si-HMGB3	5'-AAAGGCAGAUAAAGUGCGCUAUGAT-3'

Supplementary Table S4. Sequences of the primers used in this study

qRT-PCR		
HOXA11-AS	Forward	5'-TTCATCCCACCTTCTGTCCTTG-3'
	Reverse	5'-TCCAGAAGACCAGGCAGATG-3'
IFNL1	Forward	5'-GGAATTGGGACCTGAGGCTT-3'
	Reverse	5'-GTGTGAAGGGGCTGGTCTAG-3'
IFNL2	Forward	5'-TAAGAGGGCCAAAGATGCCT-3'
	Reverse	5'-CTCAGCCTCCAAAGCCATG-3'
HMGB3	Forward	5'-ACATCACTAAGGCGGCAAAG-3'
	Reverse	5'-TTTCCGGGCAACTTTAGCAG-3'
