# Supplementary material for: HOXA11-As Promotes Lymph Node Metastasis Through Regulation of IFNL and HMGB Family Genes in Pancreatic Cancer
Source: Int J Mol Sci. 2024 Nov 30;25(23):12920. doi: 10.3390/ijms252312920 (PMC11641524; doi:10.3390/ijms252312920)
Supplement: Supplementary file 1 [file ijms-25-12920-s001.zip › Suppl Figures_20241110.pdf]

Supplementary Figure S1

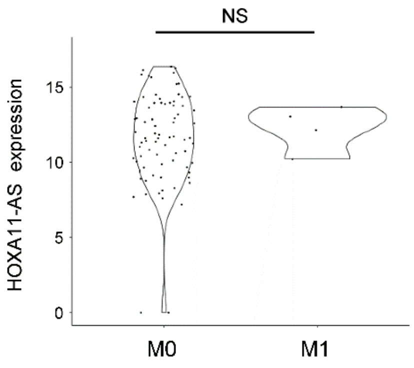

Supplementary Figure S1

Association between HOXA11-AS expression and clinical features in primary PDAC tumors. Levels of HOXA11-AS expression in PDAC tumors with the indicated M factors in TCGA dataset. NS, not significant.

Supplementary Figure S2

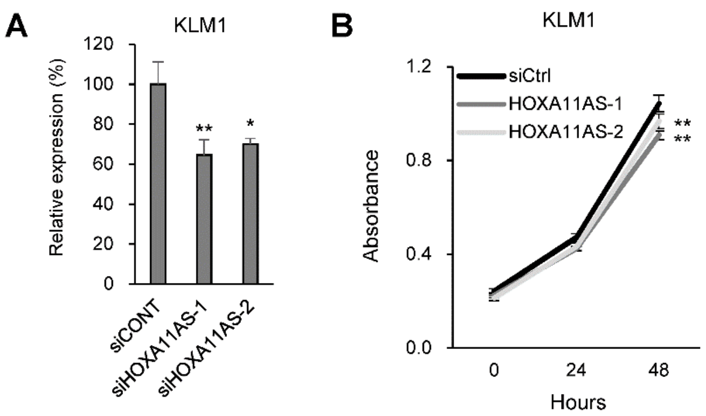

Supplementary Figure S2

Functional analysis of HOXA11-AS in KLM1 cells. (A) qRT-PCR analysis of HOXA11-AS in KLM1 cells transfected with a control siRNA or siRNAs targeting HOXA11-AS. (n = 3). (B) Cell viability assays in KLM cells transfected with the indicated siRNAs. (n = 6). Error bars represent SDs. *P<0.05, **P<0.01.
